# Deletion of the α-(1,3)-Glucan Synthase Genes Induces a Restructuring of the Conidial Cell Wall Responsible for the Avirulence of *Aspergillus fumigatus*

**DOI:** 10.1371/journal.ppat.1003716

**Published:** 2013-11-14

**Authors:** Anne Beauvais, Silvia Bozza, Olaf Kniemeyer, Céline Formosa, Viviane Balloy, Christine Henry, Robert W. Roberson, Etienne Dague, Michel Chignard, Axel A. Brakhage, Luigina Romani, Jean-Paul Latgé

**Affiliations:** 1 Unité des *Aspergillus*, Institut Pasteur, Paris, France; 2 Department of Experimental Medicine and Biochemical Sciences, University of Perugia, Perugia, Italy; 3 Molecular and Applied Microbiology, Leibniz-Institute for Natural Product Research and Infection Biology (HKI), University of Jena, Jena, Germany; 4 Integrated Research and Treatment Center, Center for Sepsis Control and Care Jena, University Hospital (CSCC), Jena, Germany; 5 CNRS, LAAS, Toulouse, France; 6 Unité de Défence Innée et Inflammation, Institut Pasteur, Inserm U874, Paris, France; 7 School of Life Sciences, Arizona State University, Tempe, Arizona, United States of America; Ohio State University, United States of America

## Abstract

α-(1,3)-Glucan is a major component of the cell wall of *Aspergillus fumigatus*, an opportunistic human fungal pathogen. There are three genes (*AGS1*, *AGS2* and *AGS3*) controlling the biosynthesis of α-(1,3)-glucan in this fungal species. Deletion of all the three *AGS* genes resulted in a triple mutant that was devoid of α-(1,3)-glucan in its cell wall; however, its growth and germination was identical to that of the parental strain *in vitro*. In the experimental murine aspergillosis model, this mutant was less pathogenic than the parental strain. The *AGS* deletion resulted in an extensive structural modification of the conidial cell wall, especially conidial surface where the rodlet layer was covered by an amorphous glycoprotein matrix. This surface modification was responsible for viability reduction of conidia *in vivo*, which explains decrease in the virulence of triple *ags*Δ mutant.

## Author Summary

*Aspergillus fumigatus* is the predominant mold pathogen of humans, responsible for life-threatening systemic infections in patients with depressed immunity. Because of its external localization and specific composition, the fungal cell wall represents a target for recognition by and interaction with the host immune cells. In *A. fumigatus*, α-(1,3)-glucan is a key component of the extracellular matrix, which encloses the cell wall β-(1,3)-glucan-chitin fibrillar core. Interestingly, the deletion of the genes responsible for α-(1,3)-glucan synthesis resulted in a mutant that exhibited wild type phenotype *in vitro*; while the altered cell wall organization resulted in this fungus being avirulent *in vivo*. This study confirms that any modification in the cell wall components is associated with compensatory reactions developed by the fungus to counteract stress on the cell wall that may result in unexpected fungal response when challenged with the host immune system.

## Introduction

α-(1,3)-Glucan is a major cell wall component of most ascomycetous and basidiomycetous fungi, including the human pathogens that establish their disease upon inhalation of their infective morphotypes (e.g., *Paracoccidioides brasilisensis*, *Histoplasma capsulatum*, *Blastomyces dermatitidis*, *Cryptococcus neoformans Aspergillus fumigatus*). The role of this polysaccharide during infection has been demonstrated and the mechanisms of its involvement in establishing virulence have been forwarded [1], [2]. In *C. neoformans*, α-(1,3)-glucan anchors the capsule, a well known virulence factor of this fungus, to the yeast cell wall and has been shown to be indirectly associated with virulence since a mutant devoid of α- (1,3)-glucan did not have any capsule and, most importantly, was unable to grow at 37°C [2]. In the yeast *H. capsulatum*, α-(1,3)-glucan was suggested to be essential for virulence because it masked immunogenic molecules: in the α-(1,3)-glucan synthase mutant, β-(1,3)-glucan that is recognized by Dectin-1, is exposed at the surface of the cell wall, whereas in the parental strain yeast cells, β-(1,3)-glucan is covered by α-(1,3)-glucan, preventing Dectin1-dependent immune response [1].

In *A. fumigatus*, α-(1,3)-glucan accounts for 40% and 19% of the mycelial and conidial cell wall polysaccharides, respectively [3]. It is a major adhesive involved in the aggregation of germinating conidia and in biofilm formation [4], [5]. Moreover, it has been shown in experimental murine aspergillosis models that α-(1,3)-glucan has a prominent immunological function conferring a long-term survival [6]. This immune protection was associated with a reduced neutrophil recruitment in the lungs and reduced inflammatory pathology [6]. α-(1,3)-glucan, like conidia, confers a Th1/Treg protection and concomitant Th2 inhibition. These *in vivo* data were confirmed by *in vitro* experiments where dendritic cells pulsed with α-(1,3)-glucan induced Il12p70 production, a classical Th1 promoting cytokine [6]. However, the physiological role of α-(1,3)-glucan could not be further investigated in absence of the mutants devoid of α-(1,3)-glucan. In *A. fumigatus*, this polysaccharide is synthesized by three α-(1,3)-glucan synthases (Agsp) [3], [7]. A triple deletion of the *AGS1*, *AGS2* and *AGS3* genes was recently generated in our lab that resulted in an *A. fumigatus* mutant lacking α-(1,3)-glucan in the cell wall. In contrast to other fungal pathogens, this triple *AGS A. fumigatus* deletion mutant did not show a distinct growth phenotype *in vitro* [8].

In the present study, three independently constructed triple *ags1*Δ*ags2*Δ*ags3*Δ (*ags*Δ) mutants devoid of α-(1,3)-glucan were used to investigate the role of α-(1,3)-glucan in *A. fumigatus* infection. As shown here, the virulence of these *A. fumigatus* triple *ags*Δ mutants was extremely attenuated in both immunocompetent and immunocompromised murine models of experimental aspergillosis tested. The defect in virulence correlated with a lack of vegetative fungal dissemination in the lungs, associated with a highly reduced inflammation following conidial inoculation. Analysis of the conidia of the triple mutants showed that the lack of virulence of the mutants *in vivo* was associated to major changes occurring on the cell wall, especially on the surface of the resting and swollen conidia, which resulted in an increased killing by phagocytes.

## Results

### The *ags1*Δ*ags2*Δa*gs3*Δ (*ags*Δ) mutants are less virulent than the parental strain in murine model of aspergillosis

**Figure 1:**
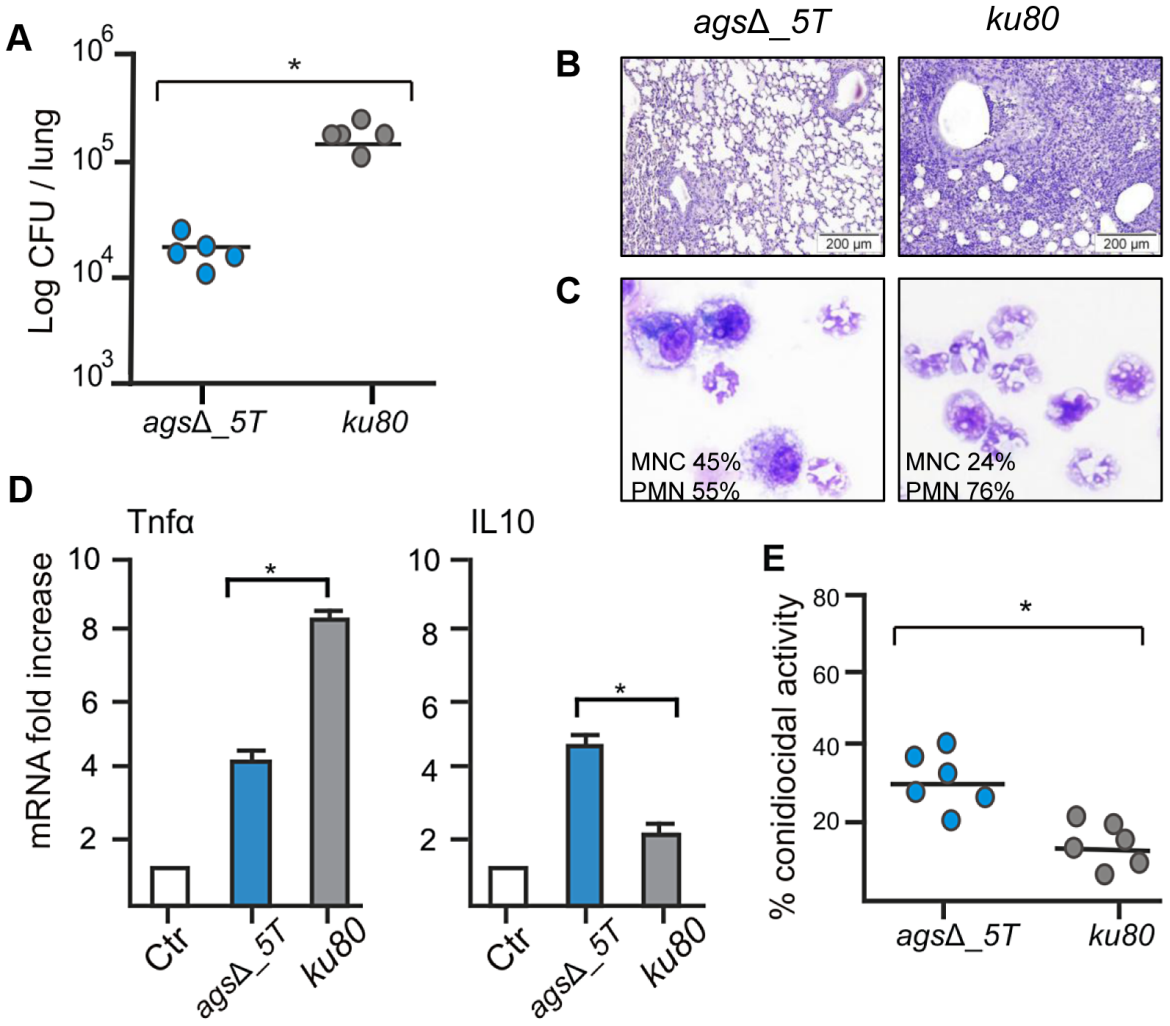
Immunocompetent mice infected with resting conidia of *ags*Δ_*5T* and parental (*ku80*) strains. Observations and analysis on mice were done four days post-infection. (A) Fungal load was expressed as log10 CFU/lung. (B) Lung histology (periodic acid-Schiff-staining). Note the polymorphonuclear cells and mononuclear infiltrates surrounding the bronchi in *ku80* infected lung. (C) After infection, percentages of monocytes and polymorphonuclear cells found in the lungs alveolar lavage (BAL). (D) Relative expression of TNFα and IL10 assessed by real time RT-PCR on lung total RNA from naïve and infected mice. (E) Conidiocidal activity by purified macrophages from uninfected mice expressed in percentage of CFU inhibition after 2 h incubation of the conidia with macrophages. Data are representative of at least three independent experiments. Ctl, naïve mice; *, P<0.05.

In the immunocompetent mice after four days of infection, the number of CFUs of the *ags*Δ mutants per lung was much lower than the CFUs per lung of the parental *ku80* strain (Fig. 1A; Fig. S1A). The reduced fungal burden of *ags*Δ was correlated to an absence of inflammation whereas a huge inflammatory response was observed with the parental strain (Fig. 1B, Fig. S1B). This was confirmed by the broncho-alveolar lavage (BAL) analysis, which showed a higher PMN recruitment after infection with *ku80* conidia compared with agsΔ (Fig. 1C, Fig. S1C). The reduced growth and inflammation in *ags*Δ infections was associated with an increase in the expression of the gene coding for the anti-inflammatory IL10 and a decreased expression of the gene coding for the pro-inflammatory TNFα in the lungs (Fig. 1D, Fig. S1D). In contrast, *ku80* infection was characterized by higher and lower expressions of TNFα and IL10, respectively.

The increased susceptibility of the *agsΔ* mutants was confirmed *in vitro* with murine alveolar macrophages isolated from BAL. After phagocytosis by the isolated macrophages, the killing of the *ags*Δ conidia was much higher than the parental strain. The resting conidia of *ags*Δ mutants were killed twice more than the parental strain after 2 h incubation with the macrophages (Fig. 1E). Further, after 6 h of incubation, the killing of the mutant reached 60–80% whereas a maximum of 30% of the parental strain conidia were killed at this time point (data not shown). Similar difference in the killing ratio between the mutant and parental strains was obtained when the conidia were pre-germinated (swollen conidia; after 6½ h incubation of the conidia in RPMI medium, at 37°C), suggesting that both resting and swollen conidia of the *ags*Δ mutants were more susceptible to conidial killing than the parental strain. This twofold increased killing susceptibility of the *ags*Δ mutants compared to parental strain did not change in the germinating morphotypes.

**Figure 2:**
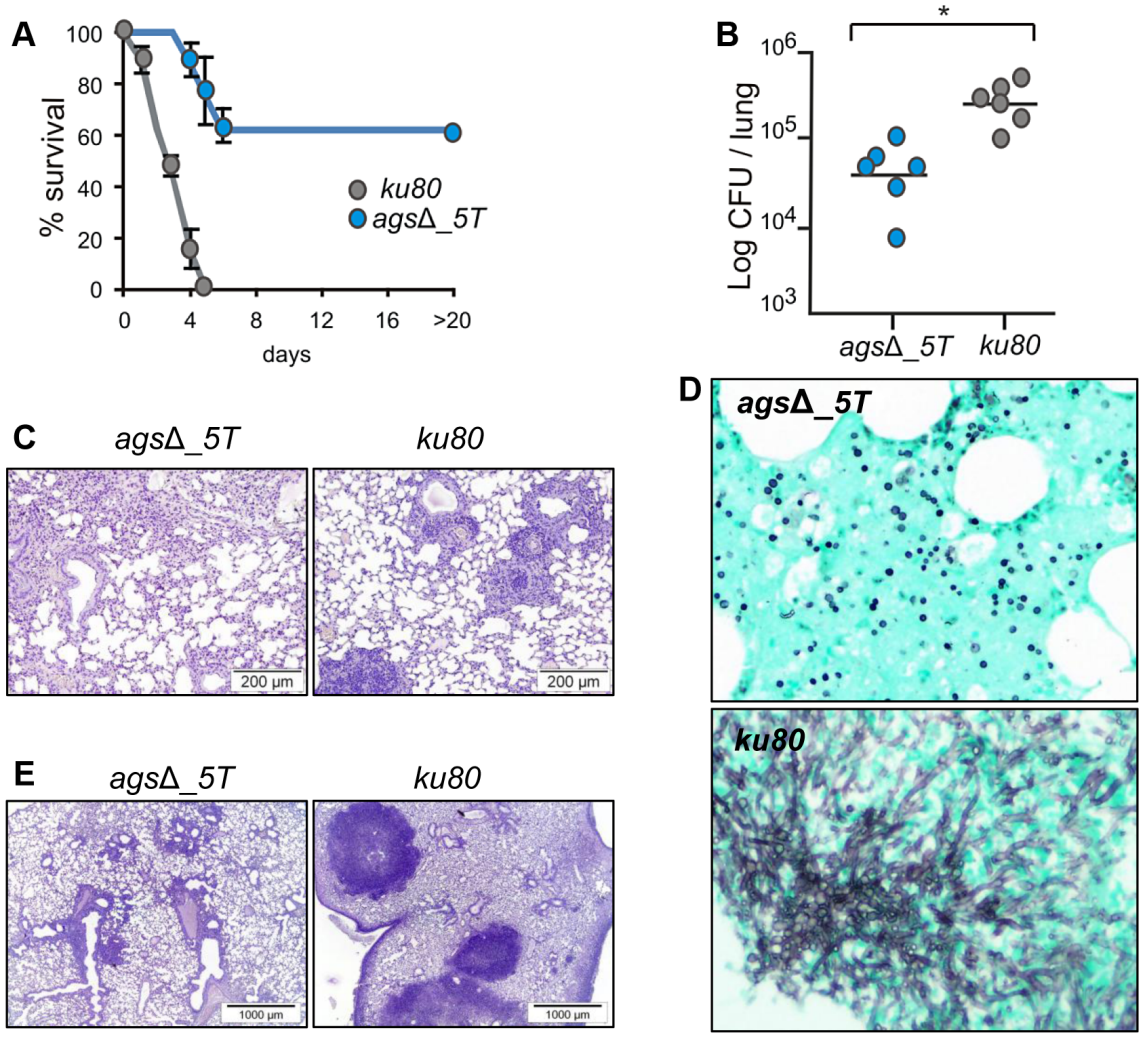
Cyclophosphamide immunosuppressed mice and anti-Ly6G treated neutropenic mice infected with resting conidia of *ags*Δ_*5T* and parental (*ku80*) strains. (A–C) Cyclophosphamide immunosuppressed mice; (D–E) anti-Ly6G treated neutropenic mice; (A) Survival (%) and (B) fungal growth estimated as CFUs in lung. (C and E) lung histology (periodic acid-Schiff-staining). Note the polymorphonuclear cells and mononuclear infiltrates surrounding the bronchi in *ku80* infected lung. (D) Histological appearance of lungs of anti-Ly6G neutropenic mice infected with conidia of *ags*Δ_*5T* and *ku80* (Gomori's methanamine silver-staining). Note the absence of mycelial development of *ags*Δ_*5T* conidia in neutropenic mice. Data are representative of at least three independent experiments. *: p<0.05.

In the experimental model of aspergillosis using immunocompromised mice, the virulence of the *ags*Δ mutants was also significantly reduced. In a cyclophosphamide model of immuno-suppression, infection with the *ku80* strain resulted in the mortality of all the mice within 4 days with a high inflammatory response, large foci of pneumonia and exudative bronchiolitis with destruction of bronchi and alveoli, whereas 60 to 80% mice infected by the *ags*Δ mutants survived and did not develop any inflammatory response (Fig. 2A–C, Fig. S2). Similar results were obtained when mice were immunocompromised by the injection of the RB6-8C5 MAb, which depletes circulating PMNs. Inhalation of the *ku80* conidia resulted in an extensive pulmonary fungal invasion with high inflammation (Fig. 2D–E). In contrast, in the RB6-8C5 MAb-treated mice lungs, only resting and swollen *ags*Δ conidia were observed and their incapability to grow vegetatively culminated in low inflammation (Fig. 2D–E). These results showed that the reduced virulence of the *ags*Δ mutant was due to a defect in their conidial survival or vegetative growth in the lung of the infected mice.

### Susceptibility of the *ags*Δ and parental strain conidia to antifungal molecules is similar

**Figure 3:**
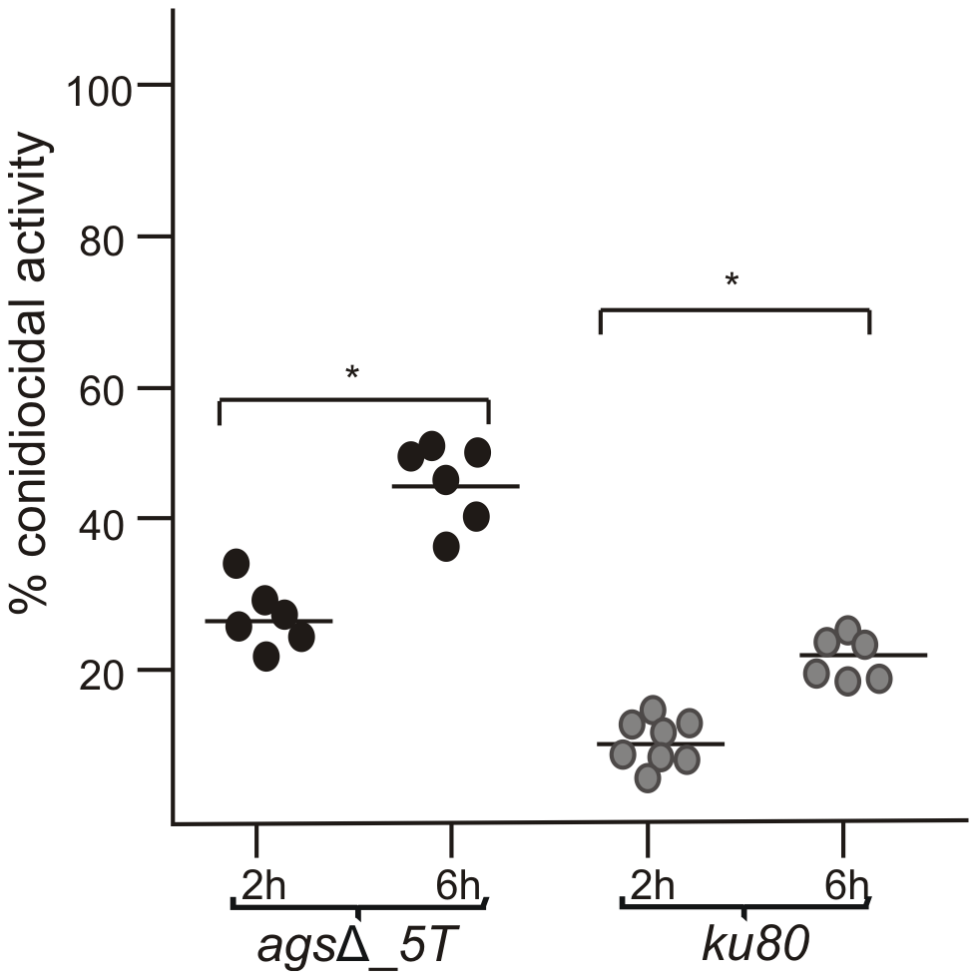
Conidiocidal activity of macrophages isolated from uninfected p47*^phox^* ^−/−^ mice against resting conidia of *ags*Δ_*5T* and parental (*ku80*) strains. Conidiocidal activity is expressed in percentage of CFU inhibition after 2 and 6 h incubation of the conidia with macrophages. Data are representative from at least three independent experiments. *, P<0.05.

To investigate the mechanisms responsible for the *in vivo* growth defect, the germination of *ags*Δ mutant conidia was tested *in vitro* under stress conditions mimicking the *in vivo* environment, such as, in the presence of reactive oxidants (ROS), cationic peptides, hypoxia and depletion of iron. The *ags*Δ mutants showed similar growth rates as their parental strain in the presence of Menadione, hydrogen peroxide and Luperox®101 with minimum inhibitory concentrations (MIC) of 30 µM, 10 mM and 2 µM, respectively (data not shown) irrespective of the pH of the medium (pH 7 or 4). The killing of resting conidia after 2–6 h of incubation with macrophages purified from uninfected p47*^phox^* ^−/−^ mice (depleted in ROS production) were similar to the killing by purified macrophages from uninfected wild type mice (C57BL6 H-2^b^) (Fig. 2B, data not shown for 6 h and Fig. 3). These results suggested that the *ags*Δ mutant conidia were not more susceptible than the parental strain conidia to reactive oxidants *in vitro* as well as *in vivo*. Interestingly, these results also suggested that in our experimental models, conidia from both mutant and parental strains were efficiently killed by ROS-independent mechanisms. Moreover, the absence of iron or the presence of a hypoxic environment did not modify the survival and conidial germination of *ags*Δ mutants compared to their parental strain (data not shown). *In vitro*, the *ags*Δ conidia germinated like parental strain conidia in culture medium without supplementation with iron as well as under hypoxic conditions (<1% (v/v) O_2_ and 9–13% (v/v) CO_2_). The *ags*Δ mutants were not more susceptible than the parental strain to cationic peptides. At doses of 230, 100, 40 and 230 µg/ml of Cathelicidin LL-37, α HNP2 and β hBD2 defensins and lactoferrin, respectively, no germination differences were seen between parental and mutant strains (data not shown). Similarly, both mutant and parental strain conidial killing was comparable with 0.05% SDS (data not shown). In addition, no increase in the intracellular labeling of the *ags*Δ mutant conidia was seen after incubation with Calcofluor White or FITC (data not shown). These results suggested that the *ags*Δ conidia were not more permeable to extracellular toxic molecules than the parental strain. Testing of these different inhibitors in combination (such as H_2_O_2_ or SDS, with Lactoferrin or LL-37) did not result in a differential sensitivity between the parental and mutant strains (data not shown).

These results suggested that, *in vitro*, the triple *ags*Δ mutants were not more susceptible to environmental stresses and antifungal molecules compared to the parental strain. To further investigate the differences in virulence between the mutant and parental strains *in vivo*, we hypothesize that the killing of the *ags*Δ mutant conidia could be due to the induction of an early and strong host immune response towards the mutant conidial morphotypes.

### The resting conidia of the *ags*Δ mutants are immediately recognized by the innate immune system because the surface rodlet layer is masked by a layer of glycoproteins

**Figure 4:**
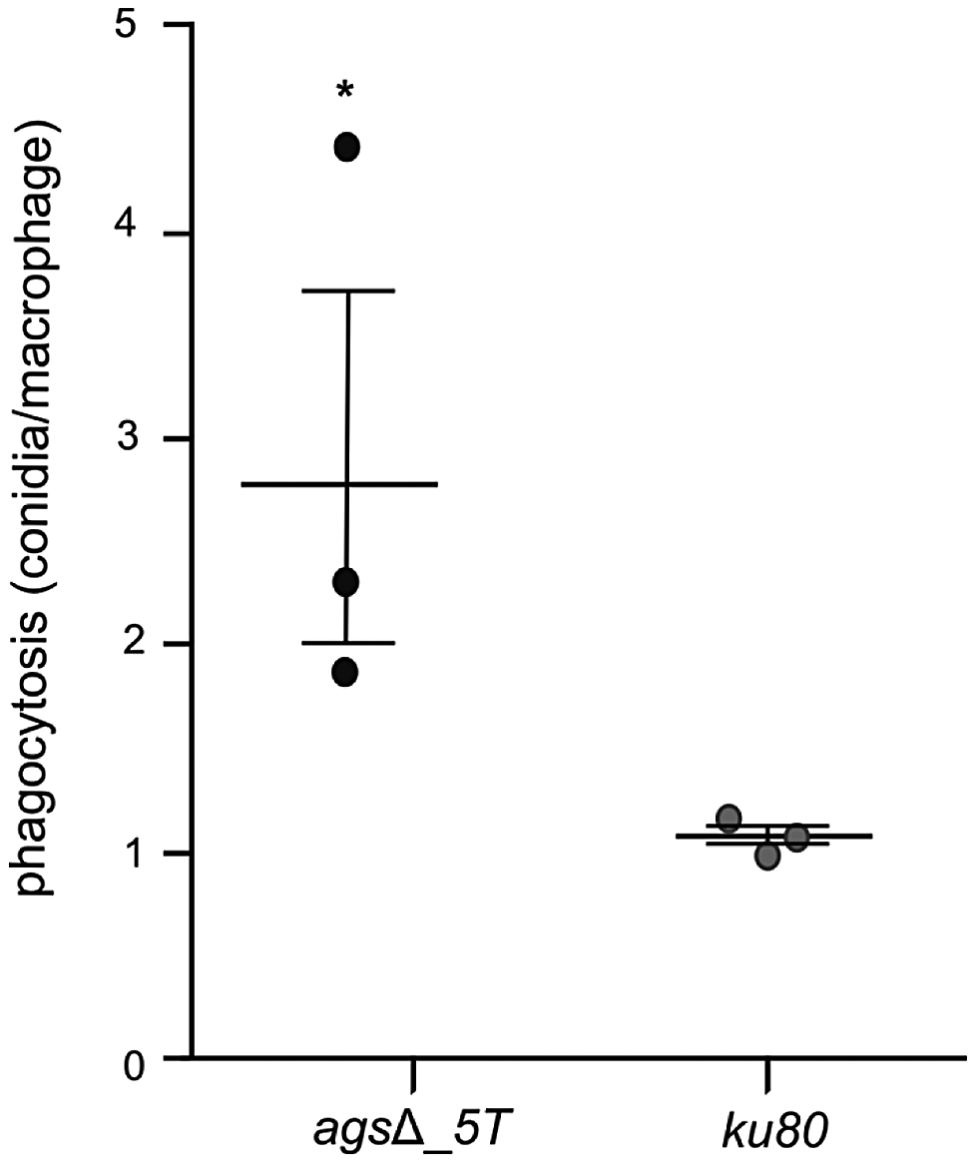
Phagocytosis activity by isolated macrophages from uninfected mice against resting conidia of *ags*Δ_*5T* and parental (*ku80*) strains. Index of phagocytosis is expressed in number of conidia per alveolar macrophage after 1*, P<0.05.

**Figure 5:**
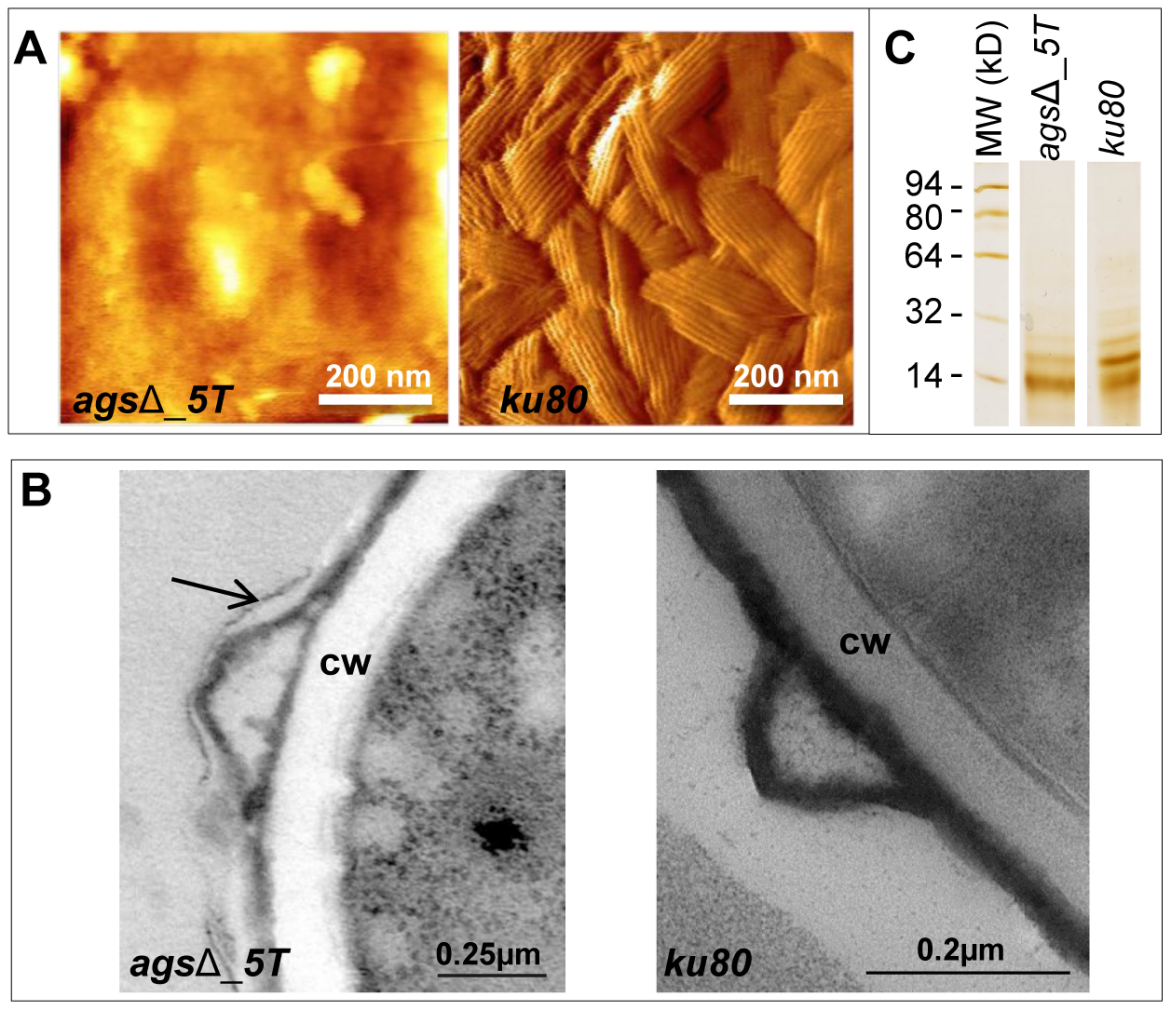
Surface analysis of resting conidia of *ags*Δ_*5T* mutant and parental (*ku80*) strains. (A): height images (z-range = 1 µm; recorded in water with silicon nitride tips). Atomic Force Microscopy (AFM) images showing the amorphous surface without the rodlet layer on the triple *ags*Δ_*5T* mutant conidia whereas the rodlet are observed on the parental strain conidial surface. (B): TEM observations. Note the presence of an extracellular material on the surface of the *ags*Δ_*5T* conidia (arrow); CW: cell wall. (C): SDS-PAGE (15% gel) of Hydrofluoric acid (HF) extracts of rodlets from resting conidia showing the two bands, 16 kDa and 14.5 kDa of RodAp classically seen from HF treatment of the conidia [10]. Data are representative of at least three independent experiments.

Resting conidia of the *ags*Δ mutant were more efficiently phagocytosed by mouse alveolar macrophages than that of the parental *ku80* strain. After 1 h incubation, an average of 3.4 and 1.4 conidia of *ags*Δ mutants and *ku80* were engulfed per macrophage, respectively (Fig. 4, Fig. S3). This result suggested that the *ags*Δ mutant and parental strain conidial surfaces are different. To investigate such structural modifications, conidial surfaces were imaged by atomic force microscopy (AFM). In contrast to the *ku80* conidia that are covered with a crystalline-like array of rodlets [9], the *ags*Δ mutant conidial surface was amorphous without any organized structure (Fig. 5A). The presence of an amorphous material covering the surface of the agsΔ conidia was further confirmed by TEM (Fig. 5B).

To investigate if the rodlet layer is still present on the *ags*Δ mutant conidial surface but masked by this amorphous material, *ku80* and *ags*Δ resting conidia were treated with hydrofluoric acid (HF) to extract the rodlet protein. Similar amount of the hydrophobic RodA protein, which constitutes the rodlet layer, could be extracted from the *ags*Δ and parental strain conidia (26.7±4.9 µg and 26.5±3.0 µg per 10^9^ conidia, respectively). Figure 5C shows that the two bands, 16 kDa and 14.5 kDa of RodAp classically seen from HF treatment of the conidia [10] were present in the SDS-PAGE profiles of *ags*Δ and *ku80* resting conidial HF-extracts. These data confirmed AFM and TEM observations that on the *ags*Δ mutant conidial surface the rodlets were present but hidden by an amorphous material.

**Figure 6:**
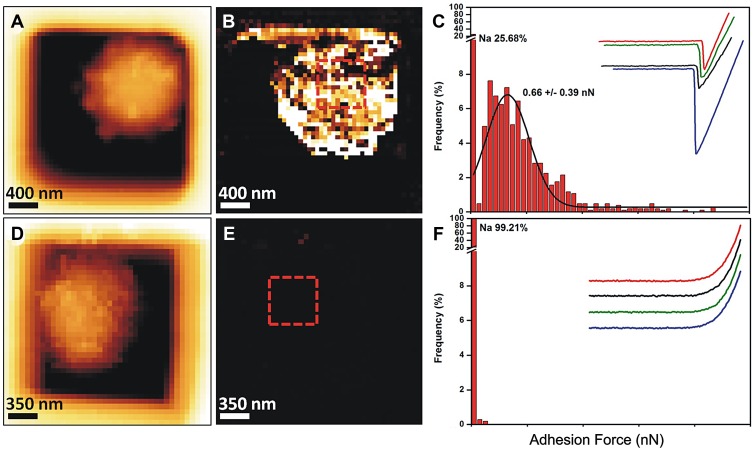
Imaging and adhesive properties of *A. fumigatus* resting conidia of the parental strain and *ags*Δ_*5T* mutant. Structural changes of *ags*Δ_*5T* correlate with a loss of cell surface adhesive properties. (A–C) parental strain; (D–F) *ags*Δ_*5T* mutant; (A, D) height images (z-range = 1 µm; recorded in water with silicon nitride tips); (B, E) adhesion force maps (z-range: 5 nN) corresponding to the height image; (C, F) Representative force-distance curves and adhesion force histograms (n = 1024) recorded on the surface of parental strain (C) and *ags*Δ_*5T* (F).

Because of the presence of this amorphous material covering the hydrophobic rodlets, we asked whether the observed surface changes correlated with differences in conidial adhesive properties. To understand this, we mapped and quantified the nanoscale adhesion properties of *ku80* and *ags*Δ mutant conidia by AFM using bare Si3N4 tip. Figure 6 (and Fig. S4) showed that the presence of this unorganized material on the *ags*Δ mutant conidial surface was associated with a dramatic reduction in their conidial surface adhesive properties. For the parental strain, force-distance curves recorded across the cell surface revealed large adhesion forces, with a magnitude of 0.6±0.039 nN as shown by the adhesion force histogram (Fig. 6A–C). In contrast, structural changes in *ags*Δ conidia caused profound modifications of the cell surface physico-chemical properties (Fig. 6D–F, Fig. S4). Force-distance curves showed the absence of adhesion forces over the entire surface of the mutant conidia. This decrease in the *ags*Δ conidial adhesion capacities indicated a modification of the cell surface hydrophobicity that could have influenced conidial phagocytosis.

**Figure 7:**
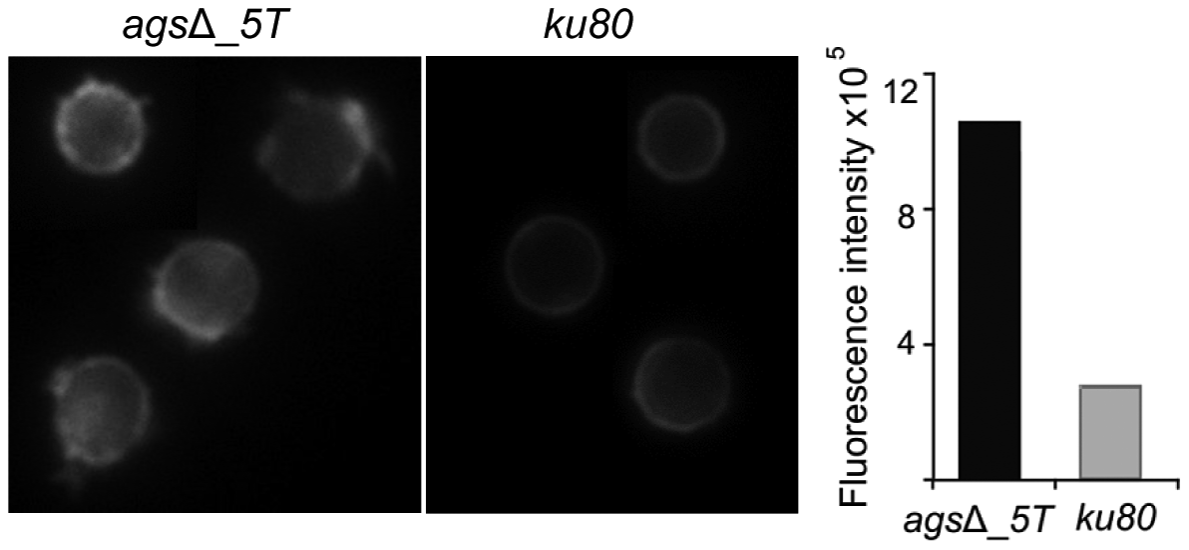
ConA-FITC labeling of *ags*Δ_*5T* mutant and parental strain (*ku80*) resting conidia. Note the increase in the ConA labeling on the *ags*Δ_*5T* mutant conidial surface. Histograms represent the calculated fluorescence intensity of the corresponding images, expressed in Einstein per seconds.

Further, chemical nature of the amorphous layer present on the *ags*Δ mutant conidial surface was investigated. It was not composed of polysaccharides since the labeling of β-(1,3)-glucan with the β-(1,3)-glucan receptor GNBP3, chitin with WGA, galactomannan (GM) with an anti-GM monoclonal antibody and galactosaminogalactan (GAG) with an anti-GAG monoclonal antibody were negative (data not shown). In contrast, a strong labeling of the resting *ags*Δ conidium with ConA was observed suggesting that the surface layer was rich in glyco-conjugates (Fig. 7).

**Figure 8:**
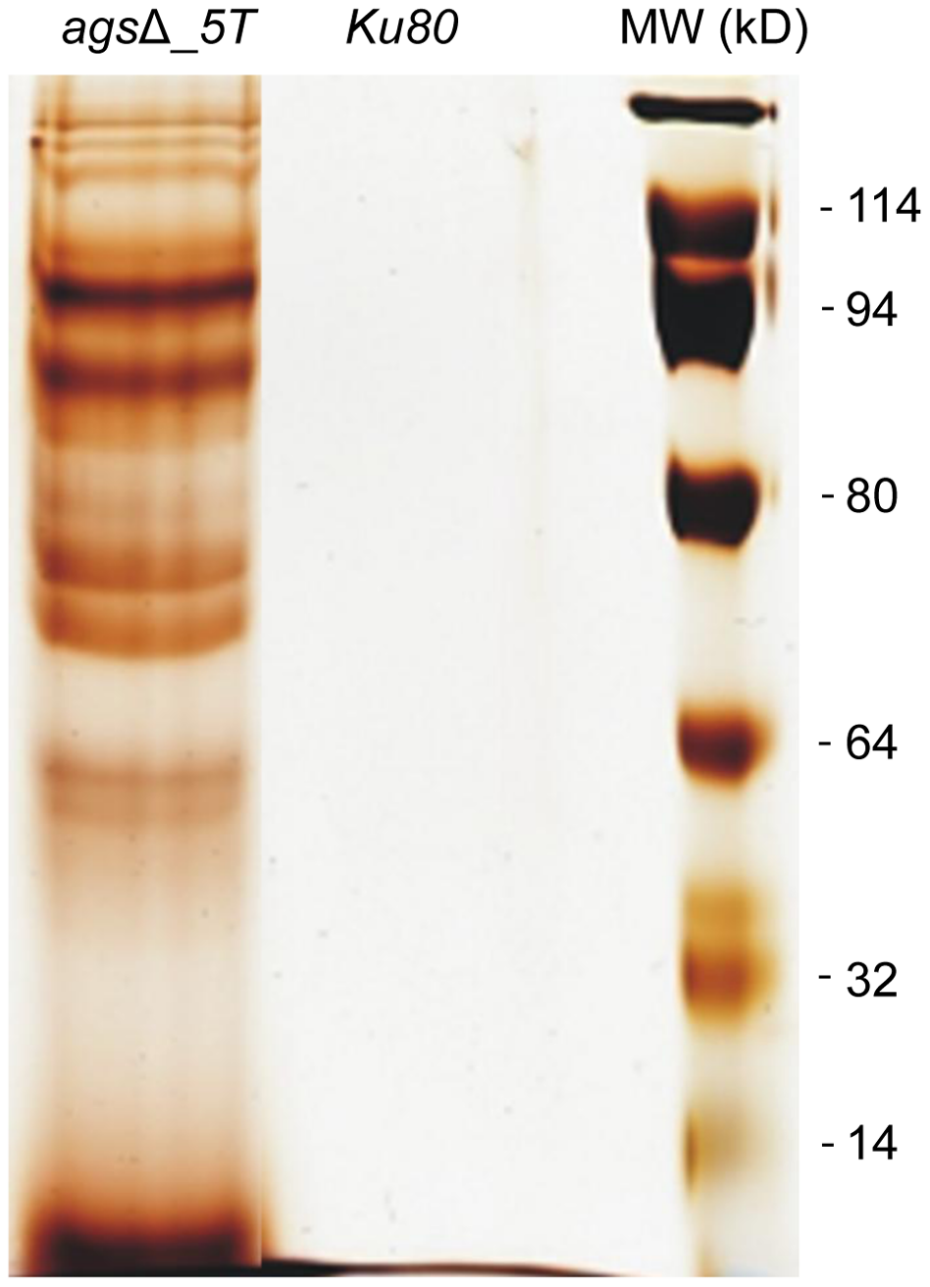
NaCl extracted proteins from the surface of *ags*Δ_*5T* resting conidia. SDS-PAGE (10% gel) of proteins extracted after 2 h incubation of *ags*Δ_*5T* and *ku80* resting conidia in 0.5 M NaCl.

To extract these amorphous surface materials, *ags*Δ resting conidia were incubated in 0.5 M NaCl for 2 h and the extracted materials were positive for protein assay. As shown in the Figure 8 (and Fig. S5), incubation with NaCl did not release any proteins from the parental *ku80* strain whereas the extracts from *ags*Δ mutant conidia contained 160 µg proteins per 10^10^ conidia. It was verified that the amorphous glycoprotein layer was removed after NaCl treatment because ConA labeling on the conidia after NaCl treatment was negative (data not shown). Further, extracted protein mixture was subjected to proteomic analysis. Thirty-four proteins were identified and in-silico analysis of these proteins by SigPred (http://www.cbs.dtu.dk/services/SignalP/) and CADRE (http://www.cadre-genomes.org.uk/Aspergillus_fumigatus/) revealed that all of them had a signal peptide except Sod1 (AFUA_5G09240, [11]) (Table 1, Table S1). Most of these proteins were hydrolases and the most abundant protein was a putative β-(1,4)-glucan hydrolase (AFUA_7G06140). Other glycosylhydrolases were hexosidases or N-acetylhexosaminidases (AFUA_1G05770; AFUA_1G14560, AFUA_1G10790, AFUA_8G05020, AFUA_6G10730). A unique aspartic phosphatase was identified that was different from the one previously identified as a major mycelial cell wall protein [12]. Three peptidases (AFUA_2G03510, AFUA_4G03490, AFUA_8G04120) and the two aspartic proteases, Pep1p and Pep2p (AFUA_5G13300, AFUA_3G11400), known to be associated with the conidial cell wall were found [13]. Two well known allergens of *A. fumigatus* were also detected (Aspf1 (AFUA_5G02330) and Aspf13 (AFUA_2G12630) [14]). Other protein such as oxidoreductases and enzymes of sugar metabolism (pyruvate dehydrogenase kinase AFUA_2G11900 and isopropylmalate dehydrogenase AFUA_1G15780) were present in lower amount as they were identified only once or twice in the proteomic survey. Interestingly, Sod1p and RodAp (AFUA_5G09580), known to be highly expressed in resting conidia [11], were also found in this NaCl extract. A similar SDS-PAGE profile was obtained when urea/thiourea buffer was used to extract *ags*Δ conidial surface material, indicating that the proteins recovered were not depending on the extraction buffer (data not shown). The fact that many proteins were present above the surface rodlet layer suggested that in contrast to the parental strain, the lack of α1,3 glucan has led to a different cell wall retainment of these glycoproteins in the *ags*Δ mutant conidia.

**Table 1:** Proteins identified in the NaCl extract of *ags*Δ_*5T* and *ags*Δ_*n8* conidia.

AFUA number	Common Name of Target	Known Gene	MW (Kd)
AFUA_7G06140	Putative secreted 1,4-β-D-glucan glucanhydrolase		78.38
AFUA_6G10130	Putative N,O-diacetyl muramidase		24.64
AFUA_1G05770	β-glucosidase ExoG2	*EXOG2*	94.75
AFUA_3G07520	Exo β-1,3-glucanase		86.72
AFUA_2G01240	Putativeβ-fructofuranosidase		57.26
AFUA_1G14560	Putative α-1,2-mannosidase, MsdS		53.84
AFUA_1G10790	Putativeα-1,2-mannosidase		92.7
AFUA_8G05020	Putative secreted α-N-acetylhexosaminidase NagA		57.4
AFUA_4G01290	Glycosyl hydrolase family 75 chitosanase		25.1
AFUA_5G13300	Secreted aspartic endopeptidase Pep1	*PEP1*	41.6
AFUA_3G11400	Secreted aspartic endopeptidase Pep2	*PEP2*	43.3
AFUA_4G03490	Putative secreted tripeptidyl-peptidase TppA, SedB	*SEDB*	65.83
AFUA_2G03380	Putative alkaline serine protease		13.4
AFUA_2G03510	Putative pheromone processing carboxypeptidase Sxa2		49.75
AFUA_8G04120	Secreted serine carboxypeptidase S1	*SCP1*	61.28
AFUA_3G07030	Putative secreted glutaminase GtaA		76.15
AFUA_2G12630	allergenic cerato-platanin Aspf13, serine alkaline protease	*ASPF13*	15.94
AFUA_5G02330	allergenic restrictocin, mitogilin Aspf1	*ASPF1*	19.59
AFUA_4G03660	Putative acid phosphatase, PhoB regulated		46.1
AFUA_5G09240	Cu,Zn superoxide dismutase Sod1	*SOD1*	16.36
AFUA_3G03450	Putative oxidoreductase		58.58
AFUA_3G08070	GMC oxidoreductase		67.61
AFUA_2G04200	4-hydroxyphenylpyruvate dioxygenase, HppD	*HPPD*	45.53
AFUA_4G13000	Putative amine oxidase		119
AFUA_4G07690	Putative phosphoribosylaminoimidazolecarboxamide formyltransferase		65
AFUA_1G16420	Uncharacterized protein		58.55
AFUA_5G09580	hydrophobin RodA	*RODA*	16.17
AFUA_1G15780	Putative 3-isopropylmalate dehydrogenase Leu2A		39
AFUA_2G11900	Putative pyruvate dehydrogenase kinase		49.43
AFUA_6G07980	Putative cyclin-dependent protein kinase		36.65
AFUA_4G03630	Putative sterol 24-c-methyltransferase		42.57
AFUA_1G11000	Putative C6 transcription factor		82.26
AFUA_1G00700	hypothetical protein		150.55
AFUA_3G06520	conserved hypothetical protein		65.71

Identification was done by MS/MS and MS with a mascot score above a threshold of 54. Details are showed in Table S1.

**Figure 9:**
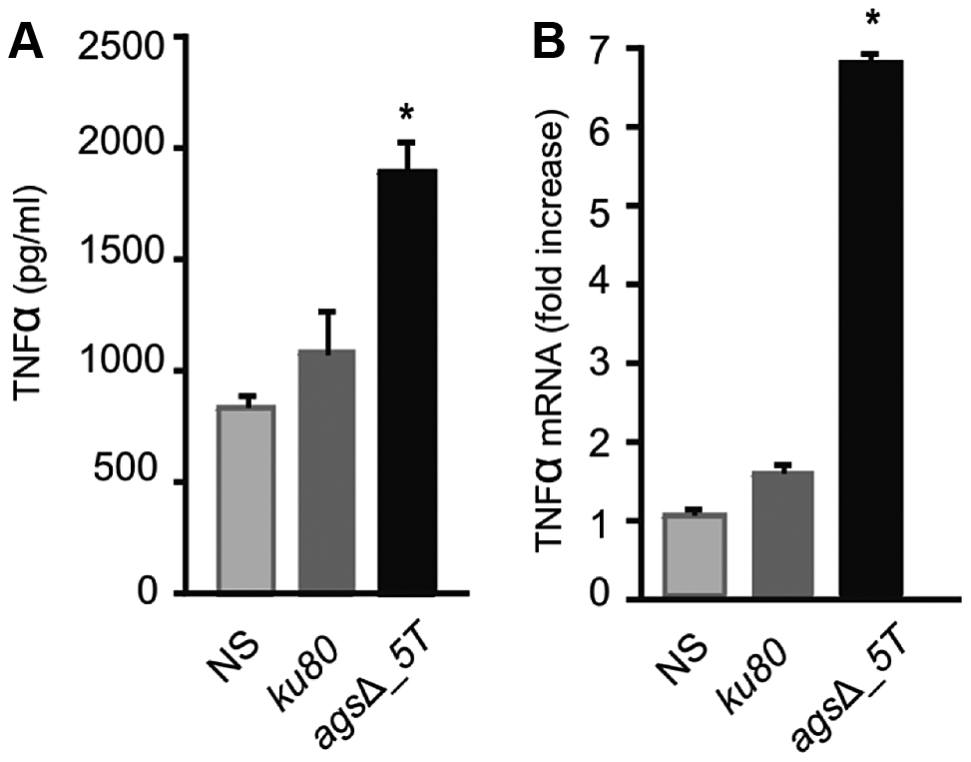
TNFα production or expression by macrophages (isolated from uninfected immunocompetent mice) upon interaction with resting conidia of parental (*ku80*) and *ags*Δ_*5T* strains or *ags*Δ_*5T* conidial NaCl extract (3.2 µg proteins) respectively. (A) TNFα was quantified after 5 h incubation of the conidia with macrophages; (B) Relative expression of TNFα assessed by real time RT-PCR in total RNA from macrophages after 5 h incubation of the *ags*Δ_*5T* conidial NaCl extract with macrophages. NaCl supernatant from *ku80* resting conidia incubated for 2 h in 0.5M NaCl was used as a control. NS: Non-stimulated. *, P<0.05.

*In vitro* analysis of the cytokines produced during the first 5 h of incubation with alveolar macrophages showed that high amounts of pro-inflammatory TNFα cytokine were produced upon interaction with *ags*Δ mutant conidia whereas no TNFα was produced when the parental strain was incubated with macrophages under the same incubation conditions (Fig. 9A, Fig. S6A). Stimulation of the macrophages with the *ags*Δ conidial NaCl extract also induced TNFα expression (Fig. 9B; Fig. S6B). These results suggested that the surface glycoprotein layer on the resting *ags*Δ conidia was responsible for the induction of pro-inflammatory cytokine production immediately after conidial phagocytosis.

Thus, the deletion of the *AGS* genes resulted in an unexpected modification of the mutant conidial surface with the emergence of an amorphous layer on the resting conidial surface over the rodlet layer, which altered biophysical properties, consequently affecting conidial interaction with the host immune system.

### Polysaccharide PAMPS are exposed on the surface of the swollen conidia of the triple *ags*Δ mutants

**Figure 10:**
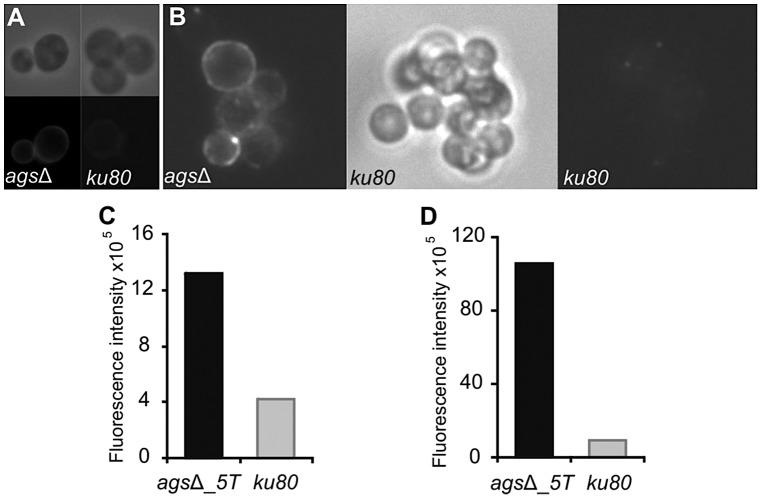
Labeling of the surfaces of *ags*Δ_*5T* and parental strain swollen conidia by WGA and the β(1,3)-glucan receptor GNBP3. The surfaces of the swollen conidia were labeled by WGA-FITC (A) and GNBP3 (B) as described in material and methods. (C, D) Histograms represented the calculated fluorescence intensity of the corresponding images (A, B respectively), expressed in Einstein per seconds.

Increased cytokine production seen in the macrophages over a 5 h-time period could also come from changes occurring at the surface of germinating conidia since it has been shown previously that conidia starts germinating intracellularly in the macrophage lysosome after the first 2 h of phagocytosis [15]. In addition, Figure 2 shows that *ags*Δ conidia undergo swelling in the infected lungs before being killed. The structural changes of the early germ tubes resulting from the *AGS* deletion were investigated by cytochemistry. The swollen conidia of the triple *ags*Δ mutants presented an increased labeling by WGA compared to the parental strain (Fig. 10A and data not shown). In addition, swollen *ags*Δ conidia were positive with the β-(1,3)-glucan receptor GNBP3, whereas both resting and swollen conidia of the parent strain were negative (Fig. 10B and data not shown). In contrast, there were no differences in the immunolabeling of the swollen conidia of parental and *ags*Δ mutants with anti-GAG and anti-GM monoclonal antibodies (Fig. S7). These results suggest that the absence of α-(1,3)-glucan that normally hides β-(1,3)-glucan and chitin, exposes these PAMPs at the surface of the swollen *ags*Δ conidia. These results were also in agreement with the chemical analysis of the cell wall: the mycelium cell wall of the *ags*Δ contained 1.7 and 2 times more chitin and β-(1,3)-glucan, respectively, than the cell wall of the parental strain [8].

**Figure 11:**
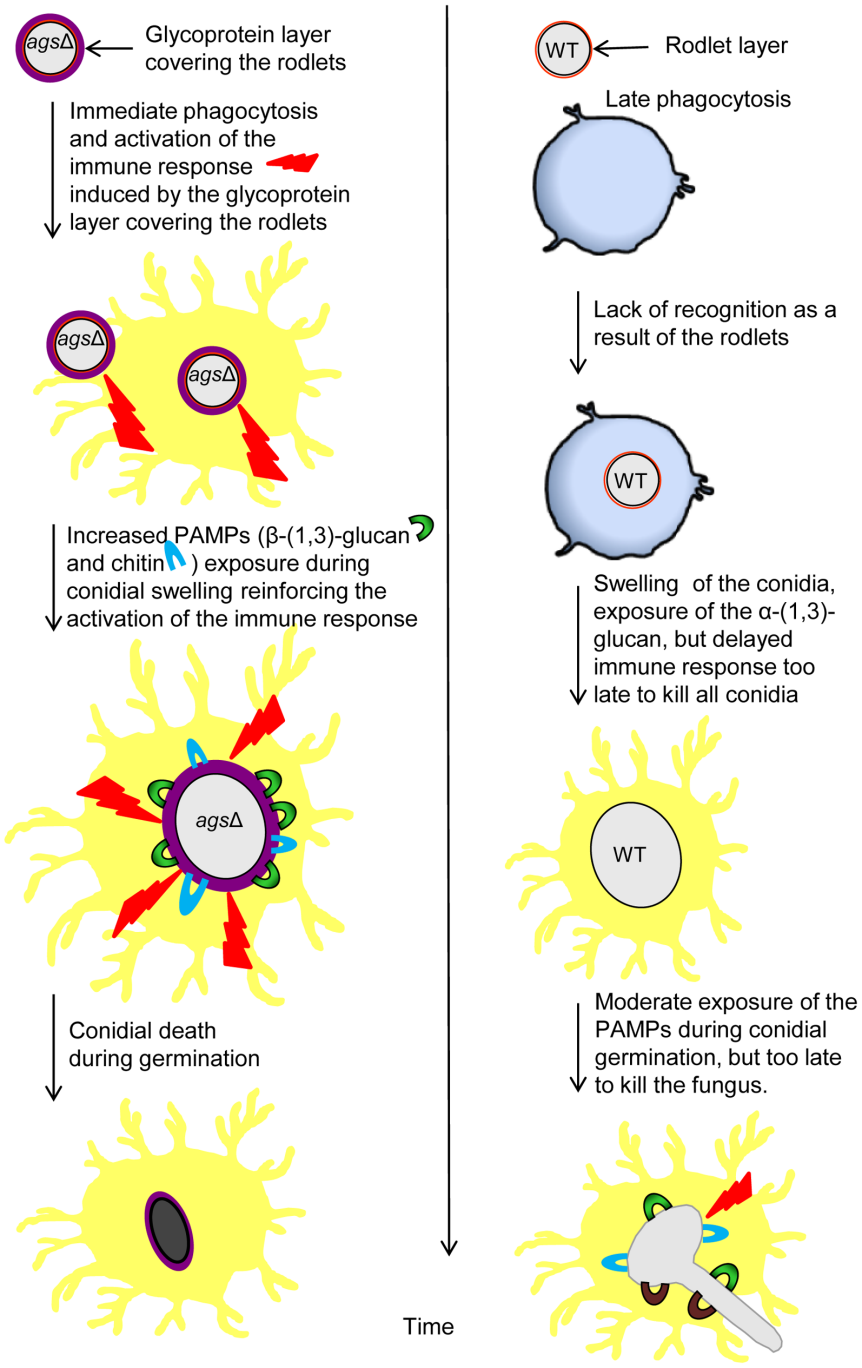
Working model explaining sequential and differential immune events upon inhalation of the *ags*Δ mutant and the parental (*ku80*) strain conidia. The presence of the glycoprotein layer on the triple *ags*Δ mutant conidial surface hides the rodlet layer. Increased exposure of PAMPs (WGA and ConA positive molecules and β-(1,3)-glucans) during vegetative growth in the triple *ags*Δ mutant modifies the host immunological response. This facilitates phagocytosis and killing of the triple *ags*Δ mutant and stimulates pro-inflammatory immune responses.

Figure 11 represents a model to explain the sequential immune events upon inhalation of the *ags*Δ mutant and parental strain conidia and their differential impact/*in vivo* fate based on our *in vitro* assays as well as *in vivo* experiments using murine aspergillosis models. The presence of glycoproteins hiding the rodlet layer increases the phagocytic rate and promotes an immediate host immunological response towards the triple *ags*Δ mutants during phagocytosis. Once the mutant conidium is internalized, the conidial swelling results in an increased exposure of PAMPs on the swollen *ags*Δ conidial surface. Such surface modifications further boosts pre-existing host defense induced by the resting *ags*Δ conidia. In contrast, the resting conidium of the parental strain are not recognized by the phagocytes and do not display major PAMPs on the surface of the conidium during the intracellular swelling. Since *ags*Δ conidia did not seem more sensitive to host antifungal molecules compared to the parental strain, we hypothesize that differences in the killing in the later growth stages resulted from an early and enhanced host response induced by the modified surface of the resting *ags*Δ conidia. This early stimulation will be responsible for the killing of the germinating *ags*Δ conidia. On the contrary, in the partially immunosuppressed experimental murine models, limited and delayed killing of the parental strain conidia enables their further vegetative growth.

## Discussion

In this study we showed that the *ags*Δ mutants displayed a reduced virulence associated with an inhibition of germination *in vivo* and a reduction of the inflammatory response after 24 h infection (decreased TNFα and increased IL10 expressions and reduced recruitment of PMNs). The low level of TNFα seen with the triple *ags*Δ mutants fits with the lack of recruitment of neutrophils seen with this mutant after 24 h infection. However, during our *in vitro* experiments with macrophages incubated during 5 h with *ags*Δ or ku80 conidia, we observed the induction of pro-inflammatory cytokines. This indicated that the lack of inflammation seen at later stages of infection in mice was due to the inhibition of vegetative growth of the *ags*Δ mutants rather than a failure to stimulate inflammation. This was in agreement with the fact that *ags*Δ conidia were killed before their hyphal development.

The primary phenotype of the resting conidia of the *ags*Δ mutants was the absence of visible rodlet layer on the conidial surface. Even though the rodlets were present in the mutant conidia, their masking by a (glyco-)protein layer restored the immune sensing that is usually silenced when the rodlets are present on the surface of the wild type conidia [10], [16]. The *ags*Δ conidia were covered by proteins, which are usually secreted during vegetative growth. Most hydrolases found in the additional amorphous surface layer of the resting *ags*Δ conidia were usually identified during mycelial growth in a protein-based medium [14], [17]. How these proteins are able to cross the conidial cell wall remains an open question. Their presence on the surface is certainly due to the modifications of the cell wall integrity resulting from the three *AGS* deletions. Interestingly, in three independent HF extractions, the amount of 14.5 kDa RodAp was slightly higher than the 16 kDa RodAp (20–23% 16 kDa RodA in *ags*Δ mutants compared to 40–50% in the parental strain; Fig. 5C) suggesting that the rodlet structure of the mutant was less organized than the rodlet of the parental strain, which putatively modified the ionic strength of the hydrophobin layer in the *ags*Δ mutants [18]. Such structural modifications may affect the adherence of the hydrophilic glycoproteins to rodlets through electrostatic binding, since these proteins were easily extracted by salt. How these glycoproteins reached the surface of the cell wall is still not understood. This should not be related to changes in cell wall permeability since the *ags*Δ mutants were not more permeable to FITC or drugs that affect viability such as ROS, cationic peptides or Calcofluor White than the parental strain (data not shown). Alternatively, the hydrolases, because of their enzymatic activity, may harm the cell wall structure itself and this would help the proteins to cross the cell wall barrier. The stimulation of the expression of TNFα after incubation with macrophages (isolated from naive mice BAL) with *ags*Δ mutant conidial NaCl extract showed that these proteins located on the conidial surface were sensed first by the immune system and were able to induce an immediate immune response towards *ags*Δ conidia. It was previously shown that some of these surface proteins are recognized by T cells and can induce a Th1 protective response [6]. In particular, the secreted aspartic protease Pep1 that has been found in NaCl extract from *ags*Δ conidia conferred protection against infection, associated with a reduced neutrophil recruitment in BAL and a reduced inflammatory pathology in the lung. Hiding of the rodlet layer by an amorphous glycoprotein layer that stimulates the host response is not exclusively specific to the *ags*Δ deletion, since a similar conidial phenotype was observed on chitin synthase mutants [19], [20]. Similarly, in *B. dermatitidis*, the absence of α-(1,3)-glucan at the surface of the yeast increased the expression of W1-1 adhesin/antigen that were bound to phagocytic cells and suppressed the generation of the pro-inflammatory cytokine TNFα [21], [22].

The exposure of polysaccharide PAMPs on the surface of germinating conidia consecutively to triple *AGS* deletions also plays a role in stimulating the host innate immune response and inducing the production of antifungal molecules by the innate immune cells. The exposure of β-(1,3)-glucan at the surface of germinating *ags*Δ conidia will favor a Dectin-1-mediated host response [23]. Similarly, increased β-(1,3)-glucan exposure due to caspofungin treatment stimulated the host defense reaction against *A. fumigatus* [24], [25]. In addition, the positive binding of WGA and ConA also suggested that other receptors such as the mannose or/and chitin/N-acetylglucosamine, which are known to stimulate an antifungal response, can also be involved in this modified immune response [26]. Similar to the situation with the *ags*Δ mutants, it was shown that the lack of α-(1,3)-glucan in *H. capsulatum* also led to the unmasking of PAMPs [1]. The protective role of α-(1,3)-glucan has been also shown in *B. dermatitidis* and *P. brasiliensis* where the absence of α-(1,3)-glucan at the surface of the yeast and/or its replacement by β-(1,3)-glucan stimulated the host defense reaction [21], [27]. Recently, the masking of chitin by α-(1,3)-glucan has been shown to be essential for the virulence of the plant pathogen *Magnaporthe grisea* [28].

The molecules responsible for the killing of the *ags*Δ conidia remain unknown. However, it is clear that ROS were not responsible for the differences in killing between the *ags*Δ mutants and the parental strain conidia since the *ags*Δ mutants did not display a higher sensitivity to ROS *in vitro* and the killing of *ags*Δ conidia was similar in p47^phox−/−^ mice compared to C57BL/6 (Fig. 3). Although a link between increased oxidative response and enhanced damage to *A. fumigatus* has been repeatedly demonstrated in the past [29], [30], recent studies, especially with chronic granulomatous disease (CGD) patients, have shown that NADPH-independent mechanisms can contribute to *Aspergillus* killing as much as ROS [31], [32]. Among possible mechanisms of NADPH-independent activity, D'Angelo et al. [33] have suggested that defensins and cathelicidins, known for their role in host defense, could be responsible for *A. fumigatus* killing in CGD mice. This seems however not the case for the *ags*Δ mutants as our *in vitro* studies indicated that the *ags*Δ mutants did not show a higher susceptibility to cathelicidin LL-37 or HNP2 and hBD2 defensins. Modification of the conidial surface may also lead to an increased binding of Surfactant Proteins A and D, Mannose Binding Lectin C or Penthraxin 3 that are known to be associated to an increased phagocytosis and an activation of the complement pathway known to play a major role in the killing of *A. fumigatus* [21], [34], [35], [36], [37]. Based on our data, it remains impossible to infer the killing of the *ags*Δ mutant conidia to currently known antifungal immune defense mechanisms. It can also be postulated that the killing may be due to an early burst of unknown toxic molecules or that the killing is the result of several antifungal molecules acting synergistically [38]. Our cell wall analysis suggested also that the cell wall architecture is perturbed in the inner as well as in the outer layer and that this perturbation may result in modifications of the cell wall permeability to specific antifungal molecules [8]. These could be responsible for an increased susceptibility of the *ags*Δ mutant to the host defense molecules.

The story of *A. fumigatus* α-(1,3)-glucan remains a two-sided coin. In the wild type strain, α-(1,3)-glucan induces an anti-*A. fumigatus* response as the injection of this polysaccharide into mice was immunoprotective and obviously responsible for the production of a Th1 response that is directed against *A. fumigatus* [6]. It could be expected that their removal favors the virulence of the mutant. In reality, the opposite happens due to the reorganization of the cell wall of the resting and germinating conidia upon triple *AGS* deletions. The presence of glycoproteins hiding the rodlet layer and the exposure of PAMPs in the germinating conidia modified the immunological response of the host, which increased phagocytosis and killing of the *ags*Δ mutants, and induced pro-inflammatory cytokine production. It is the structural modification of the entire cell wall consecutive to the *AGS* deletions that is responsible for an early stimulation of the host defense reactions. Interestingly, these structural modifications did not modify the survival of the fungus *in vitro* but are essential for the *in vivo* survival. The difference in the surface composition of the resting and swollen conidia of the *ags*Δ mutants led to an immediate sensing of the immunogenic molecules resulting in an early response of the phagocyte towards the *ags*Δ conidia. The deleterious effect of a delayed immune response on the microbial virulence is well known.

The α-(1,3)-glucan study tells us that the deletion of one cell wall gene does not lead only to the disappearance of the product of the encoded gene but results in a complete restructuration of the fungal cell wall. This has been shown with the deletion of the *AGS* genes in this study but also with other cell wall genes or consecutively to the use of antifungals acting on the cell wall in several fungal species [39]. Such structural and chemical modifications in the cell wall will have an obvious impact on the immune response of the host towards the corresponding mutant. Our study also suggests, any interpretation stating that the immune response towards a cell wall mutant is only due to the lack of the product of the deleted gene should be considered with care [40], [41].

## Materials and Methods

### Strains and culture conditions

All strains were grown in 2% (w/v) malt agar slants and 1 week-old conidia were recovered from the slants by vortexing with 0.05% (v/v) Tween 20 aqueous solution. Swollen conidia and germ tubes were produced after 5 h and 10 h, respectively, after incubation at 37°C in Brian's medium (Brian) [42]

The *A. fumigatus* parental strain AkuB^ku80^ΔpyrG (*ku80*, [43]) and three *ags*Δ mutant strains independently obtained: *ags1*Δ*ags2*Δ*ags3*Δ_*5T* (*ags*Δ_*5T*) obtained previously [8] and two new ones, *ags1*Δ*ags2*Δ*ags3*Δ*n8*and *ags1*Δ*ags2*Δ*ags3*Δ_*n6.2* (*ags*Δ_*n8* and *ags*Δ_*n6.2*), were used in this study. Since it had been impossible to complement *ags*Δ mutant for reasons explained previously [8], two new triple *ags*Δ mutants were constructed independently using the strategy described previously to exclude the possibility that undesired mutations had occurred during the deletion process. The lack of α-(1,3)-glucan in the cell wall of mutant strains was confirmed by both chemical and immunolabeling assays (Fig. S8). Chemical analysis of the cell wall was performed as previously described [44]. For immunolabeling assays, 5–10 h germinated conidia were labeled using the MOPC 104E monoclonal antibody, which binds specifically to α-(1,3)-glucan [45] (Beauvais A. Institut Pasteur, Paris, France, unpublished results). Paraformaldehyde (PFA) fixed swollen and germinating conidia were permeabilized prior to immunolabeling as previously described [46]. MOPC 104E (Sigma) and control mouse IgM (Sigma) were used at a dilution of 1∶25 and the goat antimouse IgG-TRITC (H+L, Sigma) was used as the secondary antibody at a dilution of 1∶50. The three triple mutants used in this study germinated, sporulated and conidiated like the parental strain *in vitro* (data not shown, [8]).

### Analysis of the conidial surface

Conidial surface was analyzed by Atomic Force Microscopy (AFM). The sample immobilization is achieved by assembling the living conidia within the patterns of microstructured, functionalized poly-dimethylsiloxane (PDMS, Sylgard 184) stamps using convective/capillary deposition [47]. Images and force measurements were performed in deionised water, respectively in contact mode and in Quantitative Imaging (QI) mode and Force Volume (FV) mode. For both experiments we used bare MLCT AUWH cantilever (nominal spring constant 0.01 N/m) (Bruker). Single cells were first localized and imaged and then switched over to QI and FV modes to record adhesion force maps. AFM Nanowizard II and III (JPK Instruments, Berlin, Germany) were used to capture the images. The cantilevers spring constants were measured by the thermal noise method [48] ranging from 0.0160 to 0.0190 N/m. Force curves were analyzed in order to determine the adhesion force between the conidia and the AFM tip. These adhesions were plotted as bright pixels, brighter colors indicating larger adhesion values. For each strain, images that were obtained for at least three conidia from independent cultures and analyzed with different tips, were representative of the entire conidial population inside each mutant and parental strain. The results acquired on the spores were analyzed on JPK Data Processing software.

The rodlet layer was extracted from the spore surface by incubating 10^9^ dry conidia with 48% (v/v) hydrofluoric acid (HF) for 72 h at 4°C. The contents were centrifuged (10,000 rpm, 10 min) and the supernatant obtained was dried under N_2_. The dried material was reconstituted in H_2_O and an aliquot was subjected to 15% (w/v) SDS-PAGE analysis and visualized by silver nitrate staining. Bands were quantified using Image lab software (BioRad).

To analyze the components present on the surface, conidia were incubated in 0.5 M NaCl solution for 2 h at room temperature at a ratio of 10^10^ conidia per ml. The NaCl supernatant was recovered after centrifugation and directly subjected to 10% SDS-PAGE (w/v). The protein concentrations in the extracts were determined by the Coomassie brilliant blue method [49], using BioRad kit and BSA as the standard. Proteomic analysis of the NaCl extract was carried out as described previously with slight modifications [50]. A total amount of 50–100 µg protein was loaded onto IPG strips (11 cm, pH 3–7; GE Healthcare Life Sciences) by in-gel rehydration. After equilibration of the IPG strips, SDS-gel electrophoresis was carried out using Criterion AnykD TGX gels (Bio-RAD). Proteins were visualised by colloidal Coomassie staining [51]. After scanning, gel images were analysed with the software Delta 2D 4.3. (Decodon). Protein spots were excised and analysed by mass spectrometry using an ultrafleXtreme MALDI-TOF/TOF device (Bruker Daltonics).

### Fluorescence microscopy

Resting and swollen conidia were PFA-fixed (2.5% (v/v) PFA in PBS) for one night at 4°C, washed three times with 0.1 M NH_4_Cl in PBS, once with PBS and then incubated with different antibodies or lectins as described previously [52].

Galactosaminogalactan (GAG) was labeled with a monoclonal mouse antibody as described previously [53] (20 µg/ml) and a mock monoclonal antibody was used as a control. The secondary goat anti-mouse IgG-TRITC (Sigma) antibody was used at a dilution of 1∶200.

Galactomannan was labeled with a rat anti-Galactofuranose (Gal*f*) monoclonal antibody (EBA2, diluted 1∶1000, a kind gift of M. Tabouret from BioRad, Steenvorde [54]). Control Rat monoclonal antibody of the same isotype and the secondary goat anti-rat FITC (Sigma-Aldrich) antibody were used at a dilution of 1∶1000 and 1∶500, respectively.

β-(1,3)-glucan was labeled with the N-terminal β-(1,3)-glucan binding domain of *Drosophila* pattern recognition receptor, GNBP3 (homologous to Mammalian Dectin 1) at a concentration of 3 µg/ml and a polyclonal mouse antiserum against GNBP3 at 1∶200 dilution (kind gifts from A. Roussel, CNRS, Orleans and D. Ferrandon, CNRS, Strasbourg, France [55]). Goat anti-mouse IgG FITC 1∶200 diluted (Sigma) was used as secondary antibodies.

The glucosamine moiety of chitin/chitosan and mannose/glucose moieties of glycoproteins and glucans were labeled respectively with WGA-FITC and ConA-FITC (Sigma) at 0.1 mg/ml concentrations upon incubating the conidia for 15 min at lab temperature.

### Susceptibility to oxidative stress conditions, Lactoferrin, Cathelicidin LL-37, HNP2 and hBD2 defensins, absence of iron and hypoxia

Stress conditions induced by Menadione (0 to 30 µM) and 2,5-Bis(*tert*-butylperoxy)-2,5-dimethylhexane (Luperox®101) (0 to 2 mM) were tested on both parental and mutant *A. fumigatus* strains grown on agar-RPMI (RPMI 1640, Sigma without glutamine) supplemented with 1% agar (Difco), 0.3 g/1 L-glutamine and 0.1 M MOPS or MES (to obtain a pH of 7 or 4, respectively) at 37°C for 24–48 h.

Stress conditions induced by Lactoferrin 0.45–231 µg/ml (Sigma) or Cathelicidin LL-37 0.45–231 µg/ml (Sigma), SDS (0.006–0.2%; Merck) and H_2_O_2_ (0.003–0.1%; Fluka) were tested on *A. fumigatus* strains grown on Brian medium without supplementation with iron or RPMI-glutamine-MOPS medium (described above) [38]. Combinations of 0.05% SDS or 0.012% H_2_O_2_ and Lactoferrin or Cathelicidin LL37 at concentrations of 231 µg/ml were tested in the same media, as described in Clavaud *et al* [38].

Stress condition induced by HNP2 (100 µg/ml; Sigma) and hBD2 (25 µg/ml; Sigma) defensins were also tested by incubating 10^6^ conidia/ml with the defensins for 10–16 h at 37°C in RPMI-glutamine-MOPS medium.

The growth of *A. fumigatus* strains was tested in Brian medium without supplementation with iron at 37°C and under hypoxia conditions using Anaero*Gen* sachet (Oxoid), which reduces the oxygen level in a jar to below 1% that results to a CO_2_ level between 9–13%.

### Transmission electron microscopy (TEM)

Aliquots (20 µl) of concentrated conidia were placed onto a Formvar-coated nickel or gold mesh grids, which were then placed between the flat sides of two B-type brass planchets (Ted Pella Inc., Redding, CA). The grids were used as spacer creating a thin layer of cells that allows higher yields of well-frozen cells. The samples were immediately frozen with liquid nitrogen under high pressure (2,100 bar) using a Bal-Tec HPM 010 high pressure freezing machine (Bal-Tec Products, Middlebury, CT, USA). Following cryofixation, the samples were freeze-substituted at −85°C in 1% glutaraldehyde (Electron Microscopy Sciences, Washington, PA, USA) and 1% tannic acid in acetone for 72 h. After, the samples were rinsed thoroughly with three changes of fresh acetone at −85°C for a total of 45 min. Cells were infiltrated with 1% OsO_4_ in acetone for 1 h at −85°C before being slowly warmed to room temperature over 5 h. The cells were then rinsed in acetone and slowly infiltrated with and polymerized in Spurr's resin. Embedded cells were cut into serial 70 nm thick sections with an Ultracut R Microtome (Leica, Vienna, Austria) and collected on Formvar-coated copper slot grids. Sections were post-stained with 2% uranyl acetate in 50% ethanol for 5 min followed by 5 min with Sato's lead citrate [56]. The grids were carbon-coated and viewed at 80 kV using a JEOL 1200EX transmission electron microscope (JEOL USA, Inc., Pleasanton, CA, USA).

### Analysis of *ags*Δ mutant virulence

Female 8- to 10-week-old inbred C57BL6 (H-2^b)^ mice were obtained from Charles River Breeding Laboratories (Calco, Italy). Experiments were performed according to the Italian Approved Animal Welfare Assurance A-3143-01. Breeding pairs of homozygous p47*^phox^* ^−/−^mice, raised on C57BL6 background, were purchased from Harlan Laboratories and bred under specific-pathogen free conditions at the breeding facilities of the University of Perugia, Perugia, Italy [33]. Infections were performed on one model of immunocompetent mice and in two different models of invasive pulmonary aspergillosis as previously described [6]. In the first immunosuppressed model, mice were subjected to intra-peritoneal administration of cyclophosphamide (150 mg/kg body weight) one day before infection as described previously [6]. In the second immunosuppressed model, mice were treated with anti-Ly6G monoclonal antibody (clone RB6-8C5 MAb; eBienscience; 100 µg/mouse) administered intra-peritoneally one day before infection. Rat anti-*E. coli* β-galactosidase (clone GLL 113) was used as a control IgG. Treatment with the anti-Ly6G MAb is known to selectively deplete mature neutrophils, eosinophils and dendritic cells [57] and at 24 h after administration, the number of circulating neutrophils dropped to 20±12/mm^3^ compared to 1120±227/mm^3^ in controls, and the treated mice continued to be low for circulating neutrophils counts up to 5-days. Mice were monitored for survival and fungal growth (determined as colony forming unit (CFU) per organ) four days post-infection as described previously [58]. All mice underwent necropsy for histopathological observation of fungal burden in the lungs four days post-infection. For histology, sections (3–4 µm) of paraffin-embedded lungs were stained following periodic acid-Schiff (PAS) protocol. Collection of the bronchoalveolar lavage (BAL) fluid and the morphometry [% monocytes (MNC) or polymorphonuclear (PMN) cells] was performed after four days infection as previously described [6]. Total and differential cell counts were performed after staining BAL smears with May-Grünwald Giemsa reagents (Sigma) before analysis. At least 200 cells per cytospin preparation were counted and the absolute number of each cell type was calculated. Cytospin preparations were observed using a BX51 microscope (Olympus, Milan, Italy). Histology images were captured using a high-resolution DP71 camera (Olympus).

For phagocytosis and conidiocidal activity, alveolar macrophages from uninfected mice were isolated from BAL as described [15]. For phagocytosis, macrophages were incubated at 37°C with unopsonized FITC (Sigma) labeled conidia [59] at an effector to conidial ratio of 5∶1, for 1 h in RPMI medium in micro-chambers (Ibitreat). Unbound conidia were removed by washing with RPMI and cells were fixed with 3% (v/v) PFA for 1 h in PBS. After fixation, the cells were incubated with a rabbit polyclonal anti-FITC antibody (Invitrogen) diluted 1∶2000 and a secondary rabbit antibody conjugated to Alexafluor 568 (dilution, 1∶2000) (Invitrogen). This last procedure labels only cell surface-associated conidia and the ingested conidia remained unlabeled. The number of ingested conidia per macrophage was determined on 200 macrophages. For conidiocidal activity, macrophages isolated from uninfected C57BL6 (H-2^b^) and p47*^phox^* ^−/−^ mice were incubated at 37°C with unopsonized resting or swollen conidia (6½ h in RPMI at 37°C), at an effector to fungal cell ratio of 1∶10, for 2–6 hours in an ELISA plate wells. After removing the supernatant, Triton X100 (1%) was added to the wells and incubated at 37°C for 10 min to lyse the macrophages and to collect phagocytized conidia. The percentage of phagocytized conidia capable of further germination was determined by spotting phagocytized conidia (at suitable dilution) on a nutritive agar medium and counting those conidia capable of forming germ tube among spotted conidial population. We verified that the use of Triton X100 to lyse macrophage did not affect conidial germination as the percentage of germinations were similar (97±1%) for the *ags*Δ_*5T*, *ags*Δ_*n6.2*, *ags*Δ_*n8* mutants and the parental strain with or without Triton-treatment. The differences in the germination of the conidia from the stock solution used for macrophage conidicidal activity study permitted the calculation of conidiocidal activity.

For cytokine quantification, total RNA was extracted from lungs of immunocompetent mice four days post-infection, or from macrophages isolated from BAL fluid of uninfected mice and incubating with *ags*Δ NaCl extracts containing 3.2 µg proteins, for 5 h. The cytokines expressed and productions were quantified by Real-time PCR and ELISA, respectively as described previously [6].

Statistical significance was analyzed by one- or two-way ANOVA or paired t-test with Prism software (GraphPad software, San Diego, CA) and *p*-values≤0.05 were considered to be significant. Data were representative of at least two independent experiments or pooled from three to five experiments. The *in vivo* groups consisted of six mice/group and experiments were repeated at least three times. Macrophage experiments were done three times with three different batches of macrophages and conidia.

All experiments were performed using the *ags*Δ_*5T*(Figs. 1–10, Table 1, Table S1). Virulence and proteomic analyses were performed also using *ags*Δ_*n8* (Figs. S1, S2, S3, S4, S5, S6, S7, S8, Table 1 and Table S1). Major phenotypes and virulence data were verified with agsΔ_*n6.2* (Figs. S1, S5, S6, S7, S8).

### Ethics statement

Mouse experiments were performed according to the Italian Approved Animal Welfare Assurance 245/2011-B. Legislative decree 157/2008-B regarding the animal license was obtained by the Italian Ministry of Health lasting for three years (2008–2011). Infections were performed under avertin anesthesia and all efforts were made to minimize suffering.

## Supporting Information

Figure S1 **Immunocompetent mice infected with resting conidia of** ***ags*** **Δ triple mutants and parental** ***ku80*** **strain.** Observations and analysis on mice were done four days post-infection. (A) Fungal CFUs in lungs infected with conidia of *ags*Δ_*5T*, *ags*Δ_*n6.2*, *ags*Δ_*n8* and *ku80*. (B) lung histology (periodic acid-Schiff-staining) and (C) Percentages of monocytes and polymorphonuclear cells found in the lung alveolar lavage (BAL) of mice infected with conidia of *ags*Δ_*n6.2* and *ags*Δ_*n8* mutants (periodic acid-Schiff-staining and Gomori's methanamine silver-staining) (D) Relative expression of TNFα and IL10 assessed by real time RT-PCR of the total RNA extracted from the lungs of naïve and mice infected with conidia of *ags*Δ_*n6.2* and *ags*Δ_*n8* mutants and *ku80*. Data are representative of at least three independent experiments. Ctl, naïve mice; *, P<0.05.
(TIF)

Figure S2 **Survival of Cyclophosphamide immunosuppressed mice infected with resting conidia of** ***ags*** **Δ_** ***n8*** **mutant and parental** ***ku80*** **strains.** The survival is expressed in percentage. Data are representative of at least three independent experiments.
(TIF)

Figure S3 **Phagocytosis after 1 h incubation of** ***ags*** **Δ_** ***n8*** **and parental** ***ku80*** **resting conidia by the macrophages isolated from uninfected mice.** Results expressed in number of conidia per macrophages. Data are representative of at least three independent experiments. *, P<0.05.
(TIF)

Figure S4 **Imaging and adhesive properties of resting conidia of** ***ags*** **Δ_** ***n8*** **mutant.** Structural changes correlate with a loss of cell surface adhesive properties. (A) Height images (z-range = 1 µm; recorded in water with silicon nitride tips); (B) adhesion force maps (z-range: 5 nN) corresponding to the height image; (C) Representative force-distance curves and adhesion force histograms (n = 1024) recorded on the surface of *ags*Δ_*n8* mutant conidia.
(TIF)

Figure S5 **NaCl extracted proteins from the surface of the resting** ***ags*** **Δ triple mutant conidia.** SDS-PAGE (10% gel) of proteins extracted after 2 h incubation of the resting conidia in 0.5M NaCl showing that the three triple *ags*Δ mutants (agsΔ_*5T*, agsΔ_*n8*, agsΔ_*n6.2*) displayed the similar protein patterns.
(TIF)

Figure S6 **TNFα production or expression by macrophages (isolated from uninfected immunocompetent mice) upon interaction with the parental strain** ***ku80*** **,** ***ags*** **Δ_** ***n6.2*** **and** ***ags*** **Δ_** ***n8*** **resting conidia, or the** ***ags*** **Δ_** ***n8*** **and** ***ags*** **Δ_** ***n6.2*** **conidial NaCl extract (3.2 µg proteins) respectively.** (A) TNFα was quantified after 5 h macrophage-conidial interaction. (B) Relative expression of TNFα assessed by real time RT-PCR in total RNA from macrophages after 5 h incubation of the *ags*Δ_*n8* and *ags*Δ_*n6.2* conidial NaCl extract with macrophages. NaCl supernatant from *ku80* resting conidia incubated for 2 h in 0.5M NaCl was used as a control. NS: Non-stimulated. *, P<0.05.
(TIF)

Figure S7 **Immunolabeling of Galactosaminogalactan (GAG) and galactomannan (GM) on the swollen conidial surface of the triple** ***ags*** **Δ mutants and parental** ***ku80*** **strains.** Note that there is no differences in the amount of GAG (A) (labeled by an anti-GAG monoclonal antibody) and GM (B) (labeled by an anti-galf monoclonal antibody) in the triple *ags*Δ mutant and parental strains.
(TIF)

Figure S8 **Immunolabeling of α-(1,3)-glucan.** Germinating conidia were labeled with MOPC that recognises α-(1,3)-glucan, and mouse TRITC conjugated anti-IgG was used as the secondary antibody. Note the absence of labeling on the triple *ags*Δ mutants - *ags*Δ_*5T*, *ags*Δ_*n6.2* and *ags*Δ_*n8*.
(TIF)

Table S1 **Identification of NaCl extracted conidial surface proteins from the** ***ags*** **Δ_** ***5T*** **and** ***ags*** **Δ_** ***n8*** **mutants by MALDI-TOF/TOF.** ^(1)^Number of peptide peaks identified per protein.
(DOCX)
