# Correction: Deletion of the α-(1,3)-Glucan Synthase Genes Induces a Restructuring of the Conidial Cell Wall Responsible for the Avirulence of *Aspergillus fumigatus*

**DOI:** 10.1371/annotation/05c0ca66-4ed9-4c04-96c6-3addac835e04

**Published:** 2013-11-22

**Authors:** Anne Beauvais, Silvia Bozza, Olaf Kniemeyer, Cécile Formosa, Viviane Balloy, Christine Henry, Robert W. Roberson, Etienne Dague, Michel Chignard, Axel A. Brakhage, Luigina Romani, Jean-Paul Latgé

The first name of author Céline Formosa is incorrect.

The correct author name is Cécile Formosa. 

