# Supplementary figures and images for: Deletion of the α-(1,3)-Glucan Synthase Genes Induces a Restructuring of the Conidial Cell Wall Responsible for the Avirulence of Aspergillus fumigatus
Source: PLoS Pathog. 2013 Nov 14;9(11):e1003716. doi: 10.1371/journal.ppat.1003716 (PMC3828178; doi:10.1371/journal.ppat.1003716)

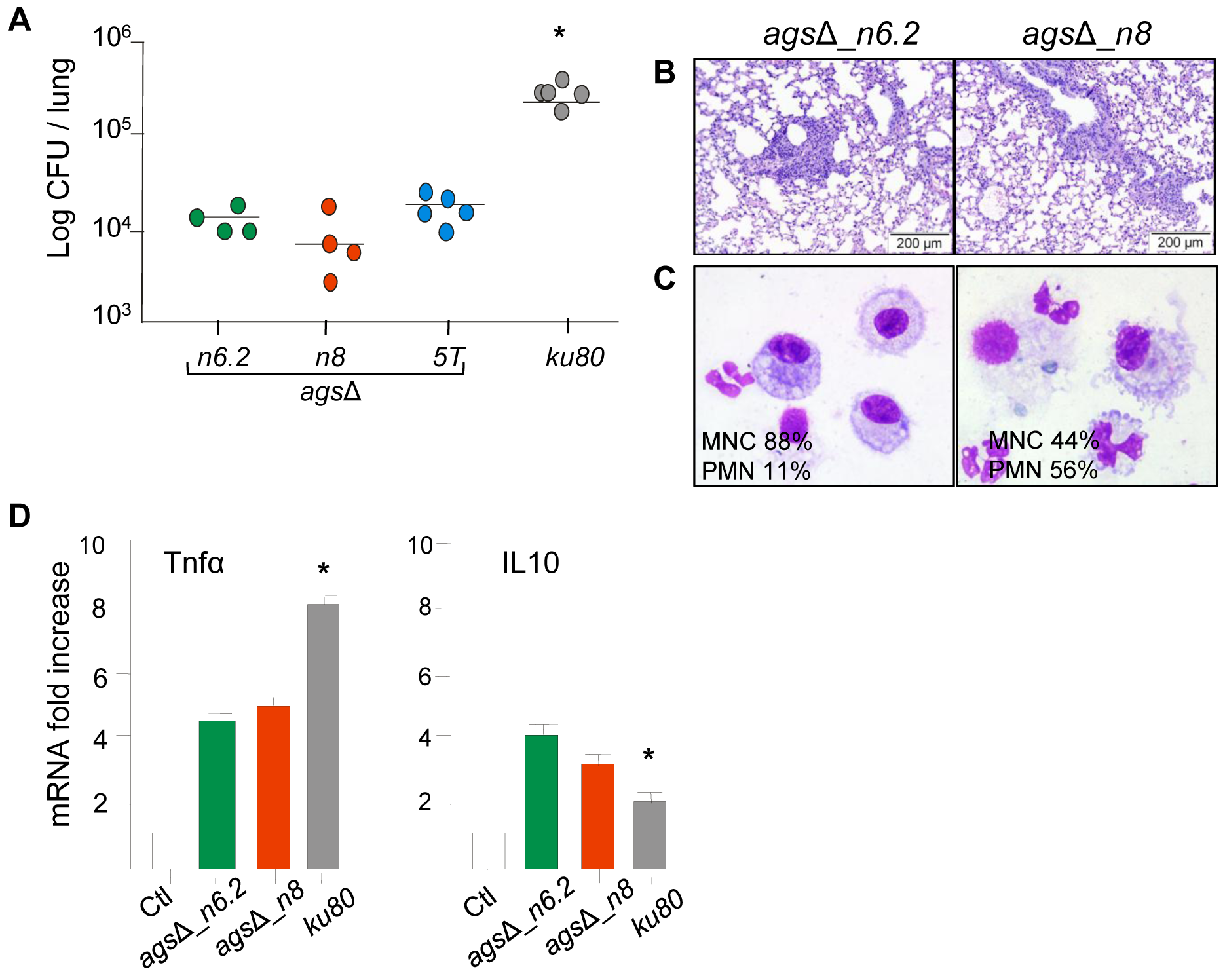

Supplement: Figure S1 — Immunocompetent mice infected with resting conidia of ags Δ triple mutants and parental ku80 strain. Observations and analysis on mice were done four days post-infection. (A) Fungal CFUs in lungs infected with conidia of agsΔ_5T, agsΔ_n6.2, agsΔ_n8 and ku80. (B) lung histology (periodic acid-Schiff-staining) and (C) Percentages of monocytes and polymorphonuclear cells found in the lung alveolar lavage (BAL) of mice infected with conidia of agsΔ_n6.2 and agsΔ_n8 mutants (periodic acid-Schiff-staining and Gomori's methanamine silver-staining) (D) Relative expression of TNFα and IL10 assessed by real time RT-PCR of the total RNA extracted from the lungs of naïve and mice infected with conidia of agsΔ_n6.2 and agsΔ_n8 mutants and ku80. Data are representative of at least three independent experiments. Ctl, naïve mice; *, P<0.05. (TIF) [file ppat.1003716.s001.tif]

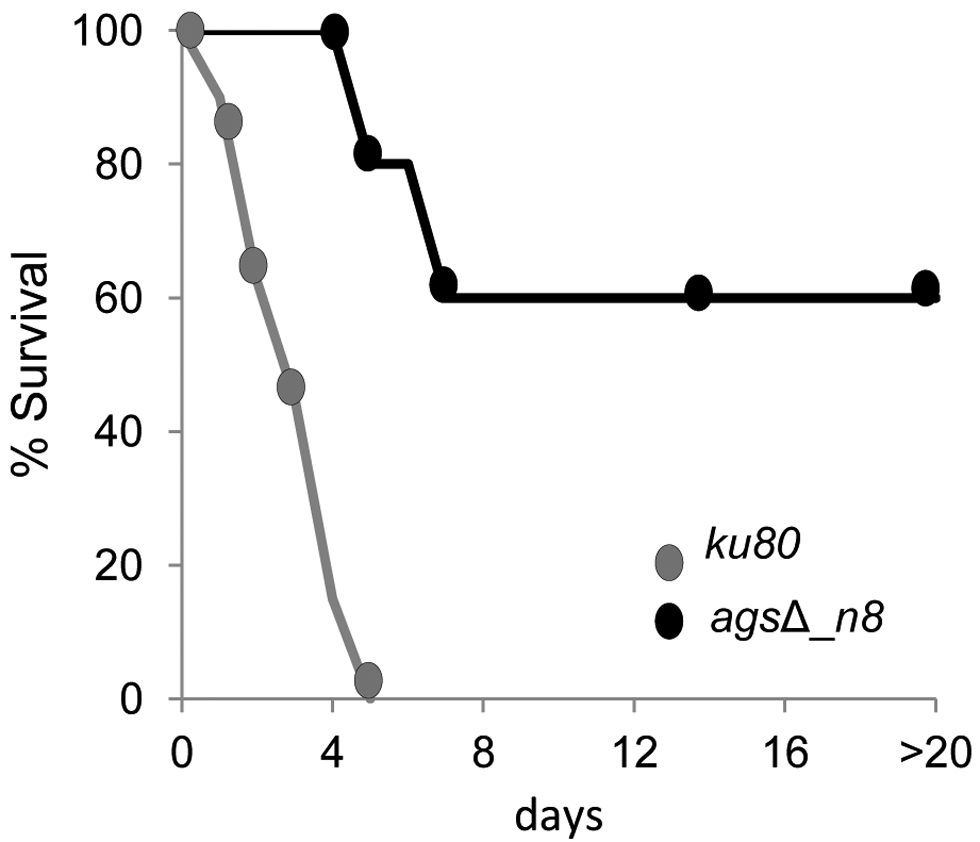

Supplement: Figure S2 — Survival of Cyclophosphamide immunosuppressed mice infected with resting conidia of ags Δ_ n8 mutant and parental ku80 strains. The survival is expressed in percentage. Data are representative of at least three independent experiments. (TIF) [file ppat.1003716.s002.tif]

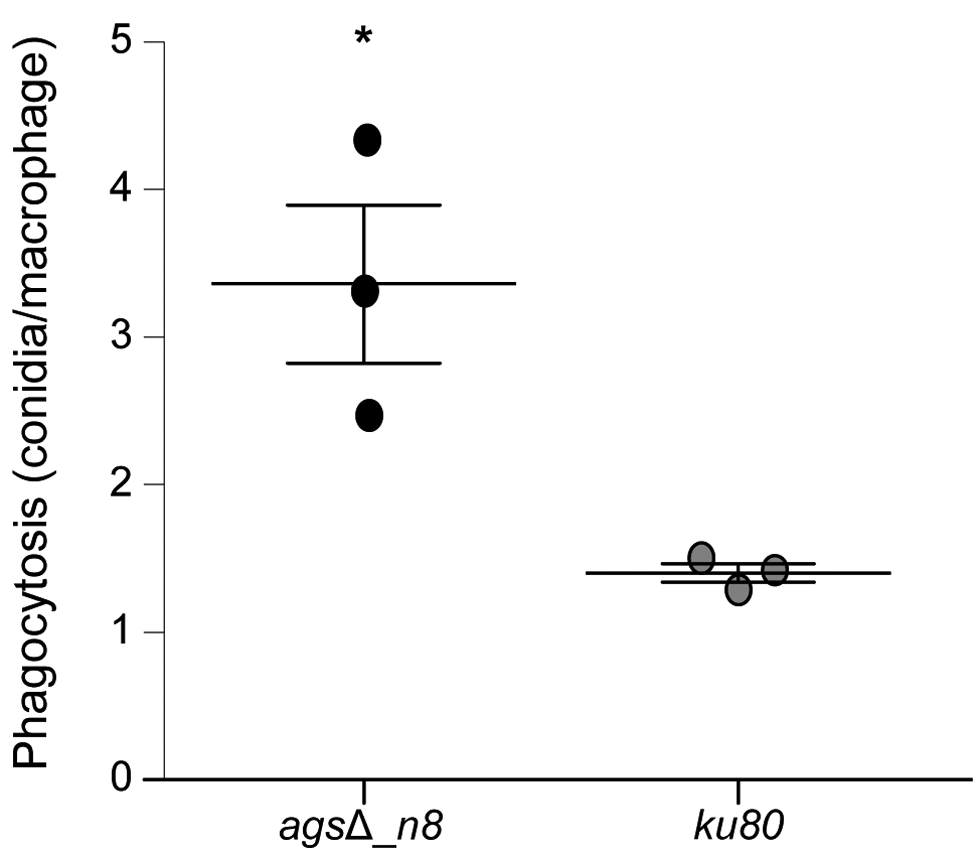

Supplement: Figure S3 — Phagocytosis after 1 h incubation of ags Δ_ n8 and parental ku80 resting conidia by the macrophages isolated from uninfected mice. Results expressed in number of conidia per macrophages. Data are representative of at least three independent experiments. *, P<0.05. (TIF) [file ppat.1003716.s003.tif]

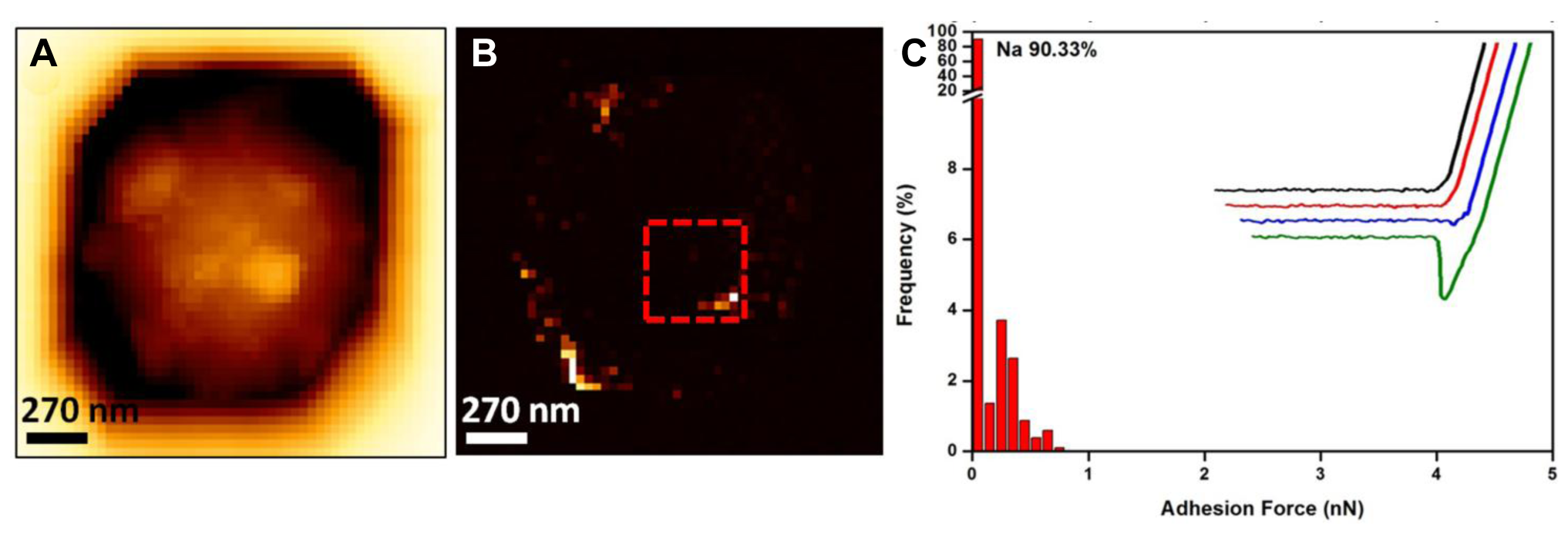

Supplement: Figure S4 — Imaging and adhesive properties of resting conidia of ags Δ_ n8 mutant. Structural changes correlate with a loss of cell surface adhesive properties. (A) Height images (z-range = 1 µm; recorded in water with silicon nitride tips); (B) adhesion force maps (z-range: 5 nN) corresponding to the height image; (C) Representative force-distance curves and adhesion force histograms (n = 1024) recorded on the surface of agsΔ_n8 mutant conidia. (TIF) [file ppat.1003716.s004.tif]

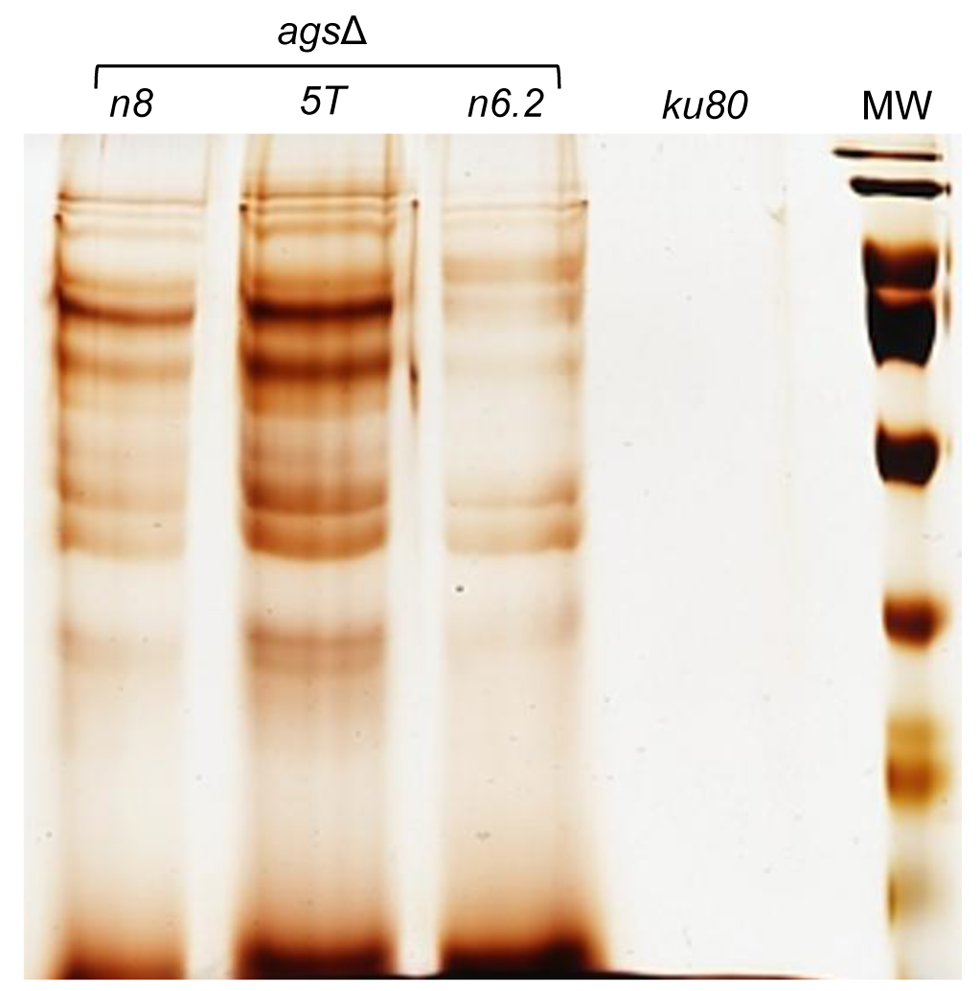

Supplement: Figure S5 — NaCl extracted proteins from the surface of the resting ags Δ triple mutant conidia. SDS-PAGE (10% gel) of proteins extracted after 2 h incubation of the resting conidia in 0.5M NaCl showing that the three triple agsΔ mutants (agsΔ_5T, agsΔ_n8, agsΔ_n6.2) displayed the similar protein patterns. (TIF) [file ppat.1003716.s005.tif]

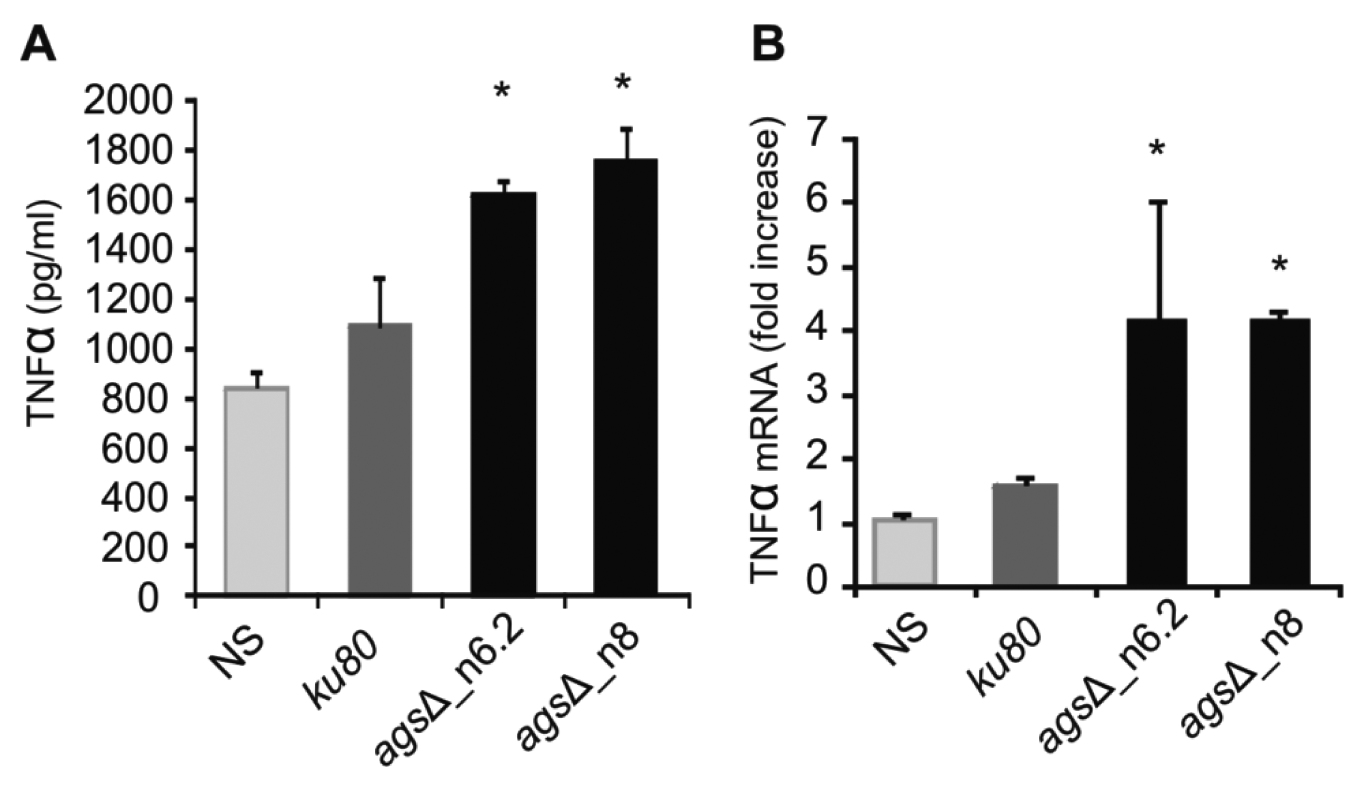

Supplement: Figure S6 — TNFα production or expression by macrophages (isolated from uninfected immunocompetent mice) upon interaction with the parental strain ku80 , ags Δ_ n6.2 and ags Δ_ n8 resting conidia, or the ags Δ_ n8 and ags Δ_ n6.2 conidial NaCl extract (3.2 µg proteins) respectively. (A) TNFα was quantified after 5 h macrophage-conidial interaction. (B) Relative expression of TNFα assessed by real time RT-PCR in total RNA from macrophages after 5 h incubation of the agsΔ_n8 and agsΔ_n6.2 conidial NaCl extract with macrophages. NaCl supernatant from ku80 resting conidia incubated for 2 h in 0.5M NaCl was used as a control. NS: Non-stimulated. *, P<0.05. (TIF) [file ppat.1003716.s006.tif]

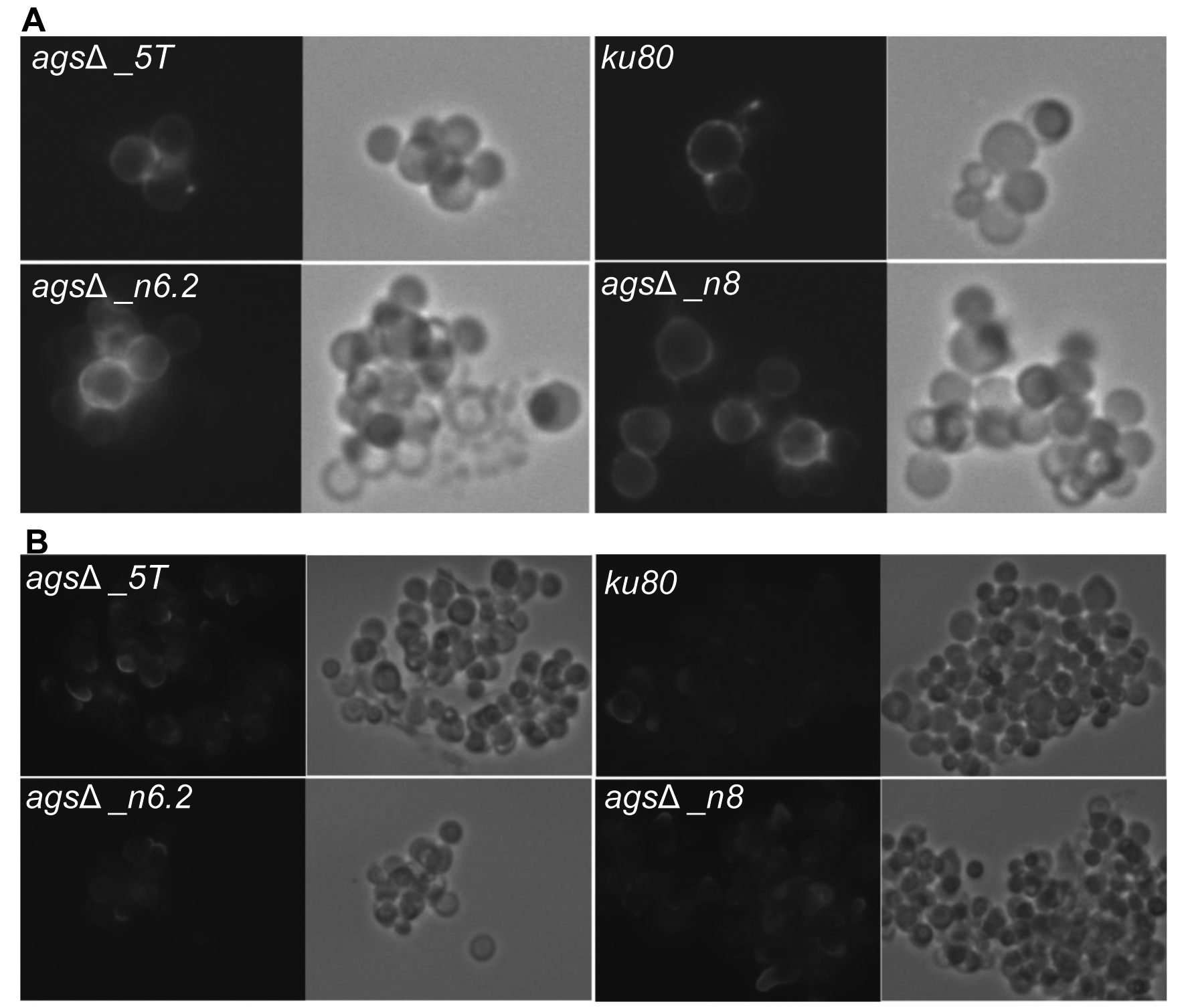

Supplement: Figure S7 — Immunolabeling of Galactosaminogalactan (GAG) and galactomannan (GM) on the swollen conidial surface of the triple ags Δ mutants and parental ku80 strains. Note that there is no differences in the amount of GAG (A) (labeled by an anti-GAG monoclonal antibody) and GM (B) (labeled by an anti-galf monoclonal antibody) in the triple agsΔ mutant and parental strains. (TIF) [file ppat.1003716.s007.tif]

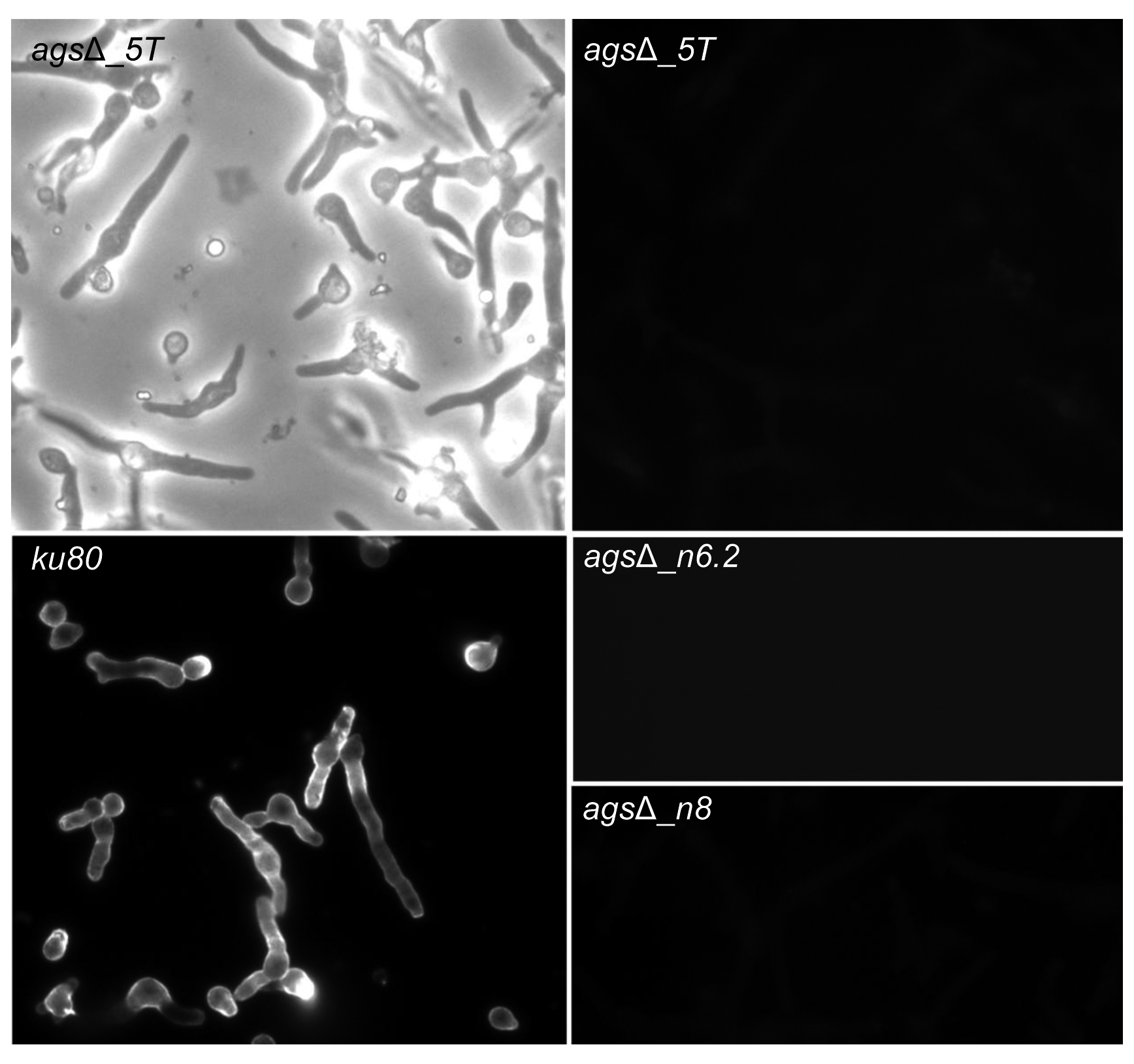

Supplement: Figure S8 — Immunolabeling of α-(1,3)-glucan. Germinating conidia were labeled with MOPC that recognises α-(1,3)-glucan, and mouse TRITC conjugated anti-IgG was used as the secondary antibody. Note the absence of labeling on the triple agsΔ mutants - agsΔ_5T, agsΔ_n6.2 and agsΔ_n8. (TIF) [file ppat.1003716.s008.tif]
