# Supplementary material for: Deletion of the α-(1,3)-Glucan Synthase Genes Induces a Restructuring of the Conidial Cell Wall Responsible for the Avirulence of Aspergillus fumigatus
Source: PLoS Pathog. 2013 Nov 14;9(11):e1003716. doi: 10.1371/journal.ppat.1003716 (PMC3828178; doi:10.1371/journal.ppat.1003716)
Supplement: Table S1 — Identification of NaCl extracted conidial surface proteins from the ags Δ_ 5T and ags Δ_ n8 mutants by MALDI-TOF/TOF. (1)Number of peptide peaks identified per protein. (DOCX) [file ppat.1003716.s009.docx]

**Table S1: Identification of NaCl extracted conidial surface proteins from the *ags*Δ_*5T* and *ags*Δ_*n8* mutants by MALDI-TOF/TOF.** ^(1)^Number of peptide peaks identified per protein.

| **AFUA number** | **Name** | **Experiment^(1)^** | **Type** | **Peptide**  **numbers** | **Mascot Score** | **Coverage [%]** |
| --- | --- | --- | --- | --- | --- | --- |
| AFUA_7G06140 | Putative secreted β-1,4- D-glucan glucanhydrolase | 10 | MS | 13 | 77,8 | 24,4 |
|  |  |  | MS/MS | 3 | 277,7 | 6,2 |
|  |  |  | MS/MS | 1 | 68,5 | 1,5 |
|  |  |  | MS | 7 | 58,2 | 14,9 |
|  |  |  | MS/MS | 1 | 81,5 | 1,8 |
|  |  |  | MS | 9 | 62,9 | 21,9 |
|  |  |  | MS/MS | 1 | 72,7 | 3,0 |
|  |  |  | MS | 8 | 56,8 | 19,9 |
|  |  |  | MS/MS | 3 | 294,9 | 8,0 |
|  |  |  | MS | 9 | 58,9 | 14,6 |
|  |  |  | MS/MS | 1 | 56,6 | 1,8 |
|  |  |  | MS | 15 | 76.0 | 25.3 |
|  |  |  | MS/MS | 2 | 202.7 | 4.7 |
|  |  |  | MS/MS | 2 | 54.5 | 4.7 |
|  |  |  | MS | 11 | 58.6 | 19.9 |
|  |  |  | MS/MS | 2 | 137.9 | 4.7 |
|  |  |  | MS/MS | 3 | 279,5 | 6,4 |
| AFUA_1G05770 | β-Glucosidase ExoG2p | 8 | MS/MS | 2 | 141,9 | 2,4 |
|  |  |  | MS/MS | 2 | 121,8 | 3,2 |
|  |  |  | MS/MS | 2 | 145,1 | 2,4 |
|  |  |  | MS/MS | 3 | 248,2 | 5,5 |
|  |  |  | MS/MS | 1 | 66,6 | 1,9 |
|  |  |  | MS/MS | 2 | 245,4 | 4,6 |
|  |  |  | MS/MS | 3 | 172,0 | 4,4 |
|  |  |  | MS/MS | 2 | 87,2 | 2,4 |
| AFUA_8G05020 | Putative secreted β-N-acetylhexosaminidase NagAp | 8 | MS | 9 | 63,3 | 15,0 |
|  |  |  | MS/MS | 1 | 118,9 | 3,0 |
|  |  |  | MS/MS | 2 | 138,6 | 5,2 |
|  |  |  | MS/MS | 2 | 182,1 | 5,5 |
|  |  |  | MS/MS | 1 | 33.6 | 3.3 |
|  |  |  | MS | 11 | 64.2 | 26.0 |
|  |  |  | MS/MS | 2 | 131.0 | 5.5 |
|  |  |  | MS/MS | 1 | 67.8 | 3.3 |
|  |  |  | MS | 12 | 73.2 | 29.7 |
|  |  |  | MS/MS | 3 | 197.9 | 8.2 |
| AFUA_4G03660 | Putative acid phosphatase, PhoBp | 7 | MS | 7 | 60,9 | 24,4 |
|  |  |  | MS/MS | 4 | 389,6 | 14,5 |
|  |  |  | MS/MS | 1 | 58,0 | 2,7 |
|  |  |  | MS/MS | 1 | 129,6 | 4,8 |
|  |  |  | MS | 8 | 78,1 | 28,0 |
|  |  |  | MS/MS | 4 | 342,1 | 11,6 |
|  |  |  | MS/MS | 1 | 56,0 | 4,8 |
|  |  |  | MS | 9 | 83,2 | 30,7 |
|  |  |  | MS/MS | 3 | 259,1 | 10,6 |
|  |  |  | MS | 6 | 65,6 | 26,8 |
|  |  |  | MS/MS | 2 | 161,6 | 8,7 |
| AFUA_2G03510 | Putative pheromone processing carboxypeptidase Sxa2p | 7 | MS/MS | 1 | 71,6 | 2,1 |
|  |  |  | MS/MS | 1 | 71,8 | 2,1 |
|  |  |  | MS/MS | 1 | 77,8 | 2,1 |
|  |  |  | MS/MS | 3 | 309,1 | 6,9 |
|  |  |  | MS/MS | 4 | 86.8 | 8.6 |
|  |  |  | MS/MS | 1 | 61.8 | 2.2 |
|  |  |  | MS/MS | 6 | 528.3 | 15.7 |
| AFUA_5G02330 | allergenic restrictocin, mitogilin Aspf1 | 6 | MS | 7 | 67,0 | 64.0 |
|  |  |  | MS | 7 | 67,0 | 64.0 |
|  |  |  | MS | 8 | 62,6 | 64.0 |
|  |  |  | MS | 8 | 84,3 | 57,0 |
|  |  |  | MS/MS | 3 | 303,9 | 23,3 |
|  |  |  | MS | 5 | 65,3 | 44.3 |
|  |  |  | MS/MS | 2 | 36.6 | 28.8 |
| AFUA_4G01290 | Glycosyl hydrolase family 75 chitosanase | 5 | MS/MS | 1 | 73.2 | 8.8 |
|  |  |  | MS/MS | 2 | 105.5 | 20.5 |
|  |  |  | MS/MS | 3 | 144.0 | 21.0 |
|  |  |  | MS/MS | 1 | 44.5 | 8.8 |
|  |  |  | MS/MS | 2 | 89.8 | 20.5 |
| AFUA_5G13300 | Secreted aspartic endopeptidase Pep1p | 4 | MS/MS | 1 | 85,5 | 3,1 |
|  |  |  | MS | 5 | 65,8 | 21.6 |
|  |  |  | MS/MS | 2 | 172.2 | 7.1 |
|  |  |  | MS | 5 | 65.8 | 21.6 |
|  |  |  | MS/MS | 4 | 374.1 | 14.2 |
| AFUA_1G14560 | Putative α-1,2-mannosidase, MsdSp | 3 | MS | 12 | 72,7 | 25,6 |
|  |  |  | MS/MS | 2 | 137,7 | 6,0 |
|  |  |  | MS | 9 | 77.8 | 23.7 |
|  |  |  | MS/MS | 3 | 168.9 | 9.7 |
|  |  |  | MS | 9 | 63.8 | 23.7 |
|  |  |  | MS/MS | 3 | 141.7 | 9.7 |
| AFUA_8G04120 | Secreted serine carboxypeptidase S1 | 3 | MS/MS | 1 | 61,8 | 2,4 |
|  |  |  | MS/MS | 1 | 79,7 | 2,4 |
|  |  |  | MS/MS | 2 | 82.4 | 6.1 |
| AFUA_4G03490 | Putative secreted tripeptidyl-peptidase TppAp, SedBp | 3 | MS/MS | 1 | 82,0 | 2,0 |
|  |  |  | MS/MS | 2 | 101,8 | 2,0 |
|  |  |  | MS/MS | 2 | 69.6 | 4.0 |
| AFUA_2G12630 | allergenic cerato-platanin Aspf13, serine alkaline protease | 2 | MS/MS | 1 | 73,0 | 7,9 |
|  |  |  | MS/MS | 1 | 85,0 | 7,9 |
| AFUA_6G10130 | N,O-diacetyl muramidase | 2 | MS/MS | 1 | 54,6 | 4,8 |
|  |  |  | MS/MS | 1 | 68.2 | 7.9 |
| AFUA_1G10790 | Putative α-1,2-mannosidase | 2 | MS | 13 | 91,7 | 22,1 |
|  |  |  | MS/MS | 3 | 234,5 | 5,8 |
|  |  |  | MS | 16 | 118,0 | 25,0 |
|  |  |  | MS/MS | 2 | 114,3 | 3,6 |
| AFUA_3G08070 | GMC oxidoreductase | 2 | MS | 7 | 60,3 | 16,5 |
|  |  |  | MS/MS | 1 | 39,7 | 1,3 |
|  |  |  | MS | 7 | 66,1 | 20,7 |
| AFUA_3G11400 | Secreted aspartic endopeptidase Pep2p | 2 | MS/MS | 3 | 232.9 | 13.8 |
|  |  |  | MS | 7 | 58.2 | 24.9 |
|  |  |  | MS/MS | 6 | 425.5 | 24.6 |
| AFUA_1G16420 | Uncharacterized protein | 1 | MS | 11 | 59,5 | 18,0 |
|  |  |  | MS/MS | 1 | 40,4 | 1,5 |
| AFUA_4G03630 | Putative sterol 24-c-methyltransferase | 1 | MS | 13 | 55,7 | 32,6 |
| AFUA_2G04200 | 4-hydroxyphenylpyruvate dioxygenase, HppDp | 1 | MS | 6 | 54,5 | 18,4 |
| AFUA_3G06520 | conserved hypothetical protein | 1 | MS | 6 | 55,9 | 14,1 |
| AFUA_3G07520 | Exo-1,3-β-glucanase | 1 | MS | 14 | 68,9 | 18,8 |
| AFUA_4G07690 | Putative phosphoribosylaminoimidazolecarboxamide formyltransferase | 1 | MS | 14 | 59,2 | 37,8 |
| AFUA_6G07980 | Putative cyclin-dependent protein kinase | 1 | MS | 7 | 54,4 | 21,7 |
| AFUA_4G13000 | Putative amine oxidase | 1 | MS | 11 | 54,1 | 13,4 |
| AFUA_1G00700 | hypothetical protein | 1 | MS | 15 | 60,6 | 12,5 |
| AFUA_5G09240 | Cu, Zn superoxide dismutase Sod1p | 1 | MS | 4 | 58.6 | 34.6 |
|  |  |  | MS/MS | 2 | 126.4 | 21.4 |
| AFUA_2G03380 | Putative alkaline serine protease | 1 | MS | 4 | 56.1 | 25.2 |
| AFUA_1G11000 | Putative C6 transcription factor | 1 | MS | 7 | 60.2 | 12.6 |
| AFUA_5G09580 | hydrophobin RodAp | 1 | MS/MS | 2 | 190.3 | 28.0 |
| AFUA_2G11900 | Putative pyruvate dehydrogenase kinase | 1 | MS | 7 | 61.0 | 25.3 |
| AFUA_3G07030 | Putative secreted glutaminase GtaAp | 1 | MS/MS | 3 | 156.6 | 6.2 |
| AFUA_2G01240 | Putative β-fructofuranosidase | 1 | MS/MS | 1 | 73.7 | 2.9 |
| AFUA_1G15780 | Putative 3-isopropylmalate dehydrogenase Leu2Ap | 1 | MS | 14 | 103.0 | 39.9 |
|  |  |  | MS/MS | 3 | 108.8 | 7.7 |
| AFUA_3G03450 | Putative oxidoreductase | 1 | MS | 9 | 54.1 | 22.2 |
